# Supplementary figures and images for: Testing the effectiveness of alcohol health warning label formats: An online experimental study with Australian adult drinkers
Source: PLoS One. 2022 Dec 7;17(12):e0276189. doi: 10.1371/journal.pone.0276189 (PMC9729007; doi:10.1371/journal.pone.0276189)

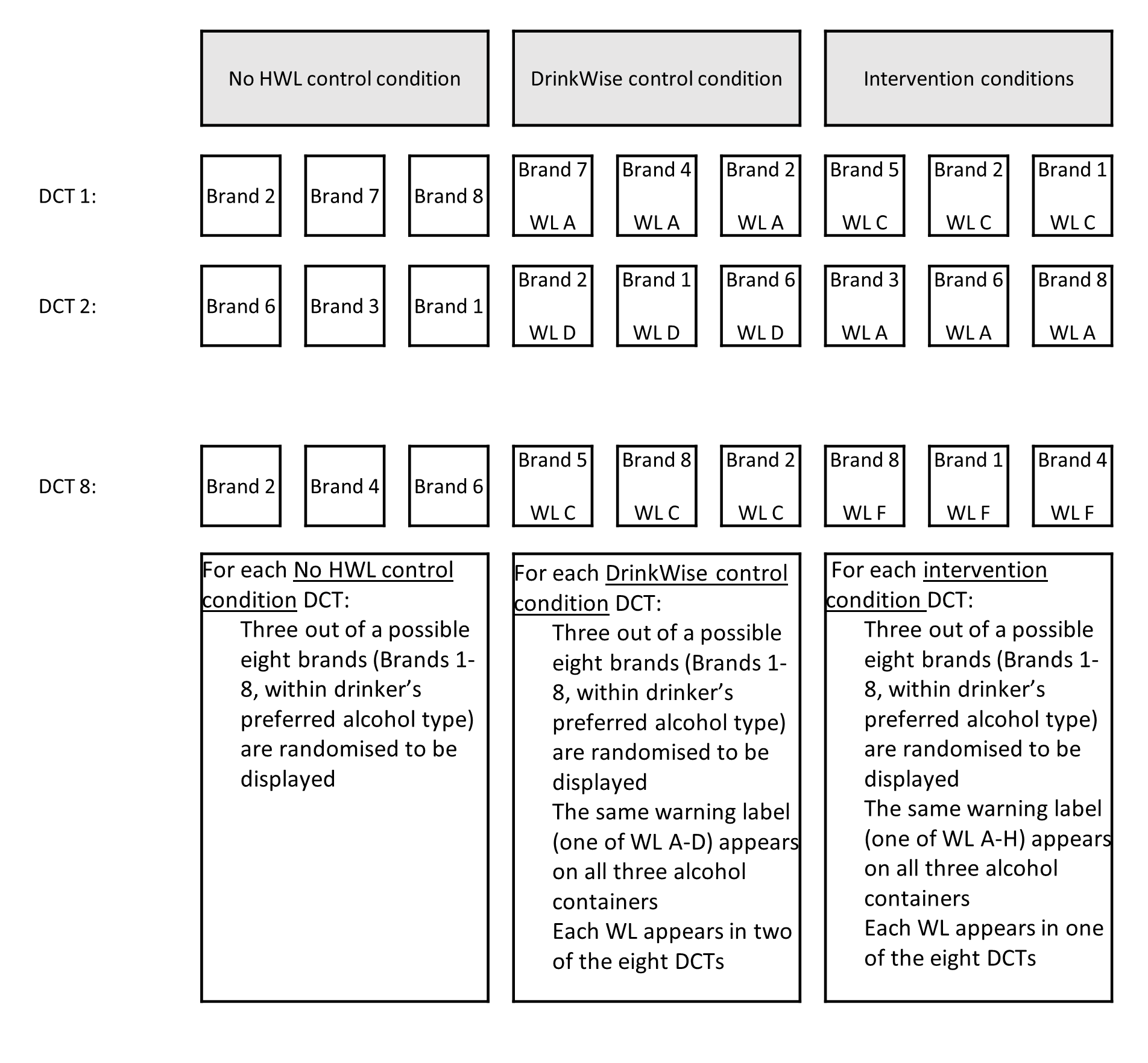

Supplement: S1 Fig — (TIF) [file pone.0276189.s001.tif]

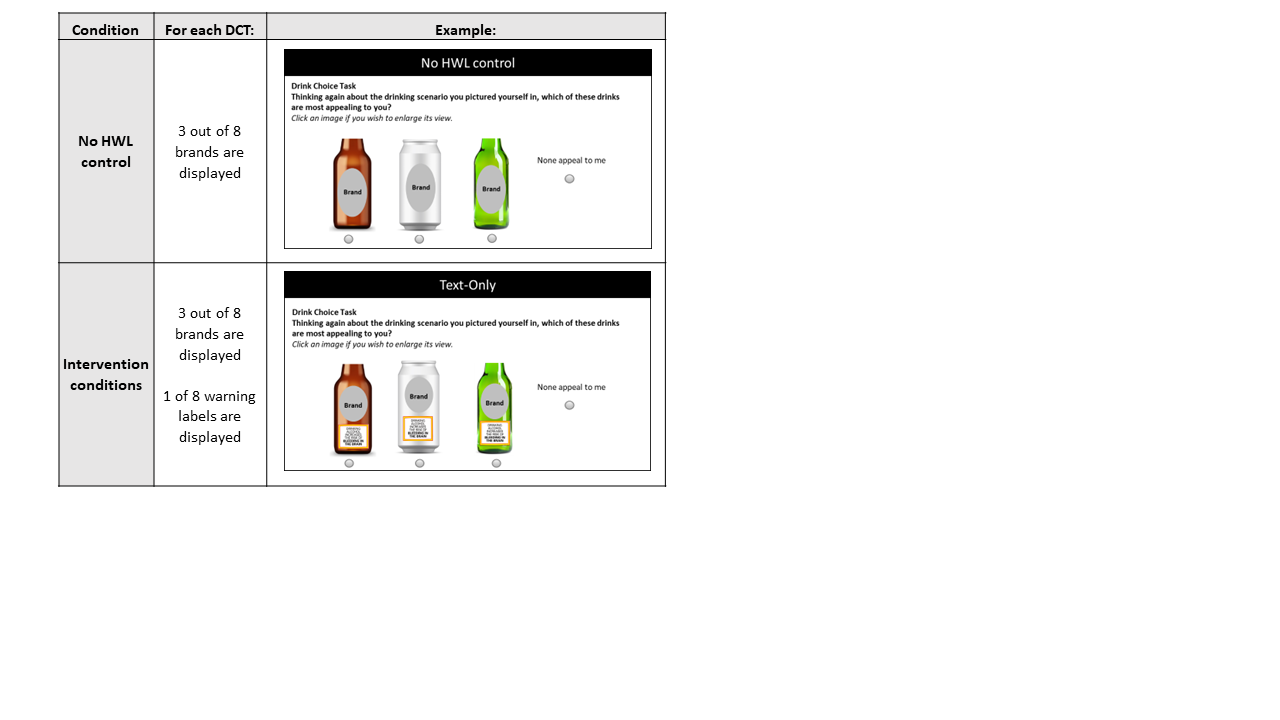

Supplement: S2 Fig — (TIF) [file pone.0276189.s002.tif]

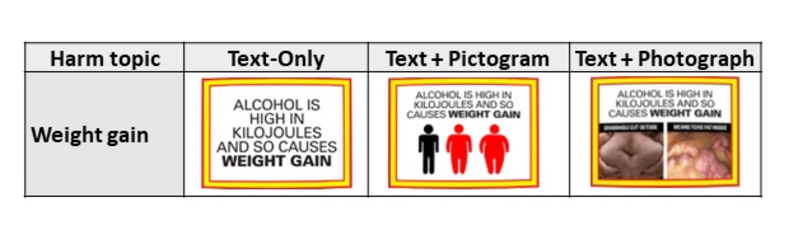

Supplement: S3 Fig — Note: We had initially pre-tested the harm topic ‘alcohol increases your risk of 8 different types of cancer’. After careful consideration and further review of the body of evidence on the causal relationship between alcohol and cancer, we changed ‘8 types of cancer’ to ‘7 types of cancer’ and removed stomach cancer from the list of cancers. While there is evidence to suggest a strong causal link between alcohol and stomach cancer, the evidence is probable and not yet convincing. (TIF) [file pone.0276189.s003.tif]
