# Supplementary material for: Testing the effectiveness of alcohol health warning label formats: An online experimental study with Australian adult drinkers
Source: PLoS One. 2022 Dec 7;17(12):e0276189. doi: 10.1371/journal.pone.0276189 (PMC9729007; doi:10.1371/journal.pone.0276189)
Supplement: S1 Table — Note. HWL = health warning label; SD = standard deviation; SE = standard error; NHMRC = National Health and Medical Research Council; LTH = long-term harm; STH = short-term harm. (PDF) [file pone.0276189.s004.pdf]

Table S1. Sample characteristics at follow-up (*N* = 1,087)

	No HWL control (*n* = 209)		DrinkWise control (*n* = 222)		Text-Only (*n* =224)		Text + Pictogram (*n* = 211)		Text + Photograph (*n* = 221)		Test
	n	**%**	***n***	**%**	***n***	**%**	***n***	**%**	***n***	**%**	***p***
**Age**											.592
18-29	94	45	97	43.7	101	45.1	96	45.5	106	48	
30-49	62	29.7	71	32	70	31.3	53	25.1	70	31.7	
50-69	53	25.4	54	24.3	53	23.7	62	29.4	45	20.4	
Mean (SD)		39.06 (14.98)		38.64 (14.68)		38.55 (14.98)		39.59 (16.54)		37.26 (15.11)	.426
**Gender**											.850
Male	98	46.9	109	49.1	112	50	101	47.9	115	52	
Not male	111	53.1	113	50.9	112	50	110	52.1	106	48	
**Educational attainment**											.487
No tertiary education or other	39	18.7	52	23.4	40	17.9	49	23.2	46	20.8	
Tertiary education	170	81.3	170	76.6	184	82.1	162	76.8	175	79.2	
**Socioeconomic status^**											.794
Low	66	31.6	64	29	64	28.6	64	30.3	58	26.2	
Mid-High	143	68.4	157	71	160	71.4	147	69.7	163	73.8	
**Parental status**											.133
No	106	50.7	124	55.9	119	53.1	113	53.6	138	62.4	
Yes	103	49.3	98	44.1	105	46.9	98	46.4	83	37.6	
**NHMRC Guidelines – LTH/STH**											.403
Low ST/Low LT risk	88	42.1	80	36	81	36.2	76	36	73	33	
High either STH LTH or Both	121	57.9	142	64	143	63.8	135	64	148	67	
**Self-perceived risky drinking**											.422
Self-perceived low-risk drinker	113	54.1	110	49.5	107	47.8	113	53.6	123	55.7	
Self-perceived high-risk drinker	96	45.9	112	50.5	117	52.2	98	46.4	98	44.3	
**Preferred alcohol type**											.107
Beer	77	36.8	74	33.3	85	37.9	70	33.2	75	33.9	
Wine	77	36.8	77	34.7	84	37.5	90	42.7	68	30.8	
Spirits	55	26.3	71	32	55	24.6	51	24.2	78	35.3	
**Past week alcohol consumption**											.999
Count (SE)	14.81 (1.27)		14.57 (0.93)		14.61 (1.10)			14.42 (1.07)		14.76 (0.96)	

Note. HWL = health warning label; SD = standard deviation; SE = standard error; NHMRC = National Health and Medical Research Council; LTH = long-term harm; STH = short-term harm

† measured at the beginning of the Drink Choice Tasks.

^ Based on national quintiles of socio-economic disadvantage where ‘Low’ is quintiles 1 and 2 (1-40%) and ‘Mid-High’ is quintiles 3-5 (41-100%).
