# Supplementary material for: Testing the effectiveness of alcohol health warning label formats: An online experimental study with Australian adult drinkers
Source: PLoS One. 2022 Dec 7;17(12):e0276189. doi: 10.1371/journal.pone.0276189 (PMC9729007; doi:10.1371/journal.pone.0276189)
Supplement: S2 Table — Note: ‘6 or more’ is listed first as it is the cut-off point we reported in our results. (PDF) [file pone.0276189.s005.pdf]

Table S2. Sensitivity analyses: frequencies and percentages of participants who completed the repeat exposure tasks for the difference cut-offs at follow-up (*N* = 1,087).

	**Number of Repeated Exposure Tasks Completed**					
	**6 or more**	**3 or more**	**4 or more**	**5 or more**	**7 or more**	**8**
No Warning Label	130 (62.2%)	170 (81.3%)	163 (78.0%)	150 (71.8%)	111 (53.1%)	71 (34.0%)
DrinkWise	141 (63.5%)	181 (81.5%)	170 (76.6%)	159 (71.6%)	121 (54.5%)	74 (33.3%)
Text-Only	137 (61.2%)	177 (79.0%)	169 (75.4%)	154 (68.8%)	113 (50.4%)	62 (27.7%)
Text + Pictogram	134 (63.5%)	179 (84.8%)	166 (78.7%)	152 (72.0%)	110 (52.1%)	67 (31.8%)
Text + Photograph	149 (67.4%)	177 (80.1%)	169 (76.5%)	160 (72.4%)	125 (56.6%)	80 (36.2%)
Full Sample	*691 (63.6%)*	*884 (81.3%)*	*837 (77.0%)*	*775 (71.3%)*	*580 (53.4%)*	*354 (32.6%)*

Note: '6 or more' is listed first as it is the cut-off point we reported in our results.
