# Supplementary material for: Testing the effectiveness of alcohol health warning label formats: An online experimental study with Australian adult drinkers
Source: PLoS One. 2022 Dec 7;17(12):e0276189. doi: 10.1371/journal.pone.0276189 (PMC9729007; doi:10.1371/journal.pone.0276189)
Supplement: S3 Table — Note. p-value for condition × dose interaction shown; and † = HC3 heteroskedasticity-consistent standard error estimator used. ‘6 or more’ is listed first as it is the cut-off point we reported in our results. (PDF) [file pone.0276189.s006.pdf]

Table S3. Omnibus test results for the condition-by-dose interaction term for different cut-offs for the completed number of repeat exposure tasks.

	Number of Repeat Exposure Tasks					
	**6 or more**	**3 or more**	**4 or more**	**5 or more**	**7 or more**	**8**
**Follow-Up**						
Past week alcohol consumption (mean)	.338	.122	.208	.201	.157	**.030**
Intentions to drink less in the next week	.471	.447	.448	.428	.847	.929
Intentions to reduce how often and/or how much consumed per occasion in the next month	.350	.753	.752	.518	.906	.956
Intentions to avoid drinking alcohol completely in the next month	.413	.714	.848	.815	.152	.069
Readiness to change (Mean)	.261	.181	.531	.394	.265	.521
Frequency of thinking about alcohol-related health risks in the past week	.804	.230	.382	.959	.613	.823
Awareness of alcohol related harms						
Increase your risk of cancer	.976	.582	.515	.671	.805	.846
Increase your risk of liver damage	.790	.185	.388	.662	.897	.482
Increase your risk of heart disease	.746	.594	.221	.375	.938	.779
Increase the risk of pregnancy complications	.959	.356	.475	.724	.979	.249
Negative emotional arousal (mean)†	**.010**	**.042**	.051	.083	.067	.052
Positive emotional arousal (mean)†	.877	.120	.081	.357	.974	.650
Show/talk about images with others	.151	.082	.192	.052	.100	.204

Note. p-value for condition × dose interaction shown; and † = HC3 heteroskedasticity-consistent standard error estimator used. '6 or more' is listed first as it is the cut-off point we reported in our results.
