# Supplementary material for: Testing the effectiveness of alcohol health warning label formats: An online experimental study with Australian adult drinkers
Source: PLoS One. 2022 Dec 7;17(12):e0276189. doi: 10.1371/journal.pone.0276189 (PMC9729007; doi:10.1371/journal.pone.0276189)
Supplement: S1 Appendix — (PDF) [file pone.0276189.s007.pdf]

Appendix S1. Regression analyses for Table 2, Table 3, Table 4 and Fig 3.

Table 2. Adjusted proportions or means, and omnibus test results for condition or condition-by-dose interaction regression analyses

```
.        /* Variables of interest:
>
>               Baseline (n=1,755)
>
>        D1_r                    // Intentions to drink less in the next week
>        D2D3_r                  // Intentions to reduce how often and/or how much consumed per occasion in the next month
>        D2_D4_4_r               // Intentions to avoid drinking alcohol completely in the next month
>
>               Follow-up (n=1,087)
>
>        fup_7day                // Past week alcohol consumption (mean)
>        FC1_r                   // Intentions to drink less in the next week
>        FC2FC3_r                // Intentions to reduce how often and/or how much consumed per occasion in the next month
>        FC2_FC4_3_r             // Intentions to avoid drinking alcohol completely in the next month
>        FC7                     // Readiness to change (mean)
>        FD1_r                   // Frequency of thinking about alcohol-related health risks in the past week
>        FD2_FD6_1_b             // Increase your risk of cancer
>        FD2_FD6_2_b             // Increase your risk of liver damage
>        FD2_FD6_4_b             // Increase your risk of heart disease
>        FD2_FD6_5_b             // Increase your risk of pregnancy complications
>        neg_ar                  // Negative emotional arousal
>        pos_ar                  // Positive emotional arousal
>        show_talk               // Show/talk images + others
>
> */


. *Declare covariates
. global covariates "i.Quota_Age_Group i.gender_A2b i.educ_G2b i.parent_G3r i.seifa_quin_b i.NHMRCrisk_b i.B17_r i.C1_b base_7day"
```

```
. /* Immediately Post-Exposure/BASELINE */
. 
. di "Intentions to drink less in the next week"
Intentions to drink less in the next week

. 
.         ** Logistic regression
. 
.         *** Simple model
.         logistic D1_r i.condition ///
>                 $covariates , ///
>                 vce(robust)

Logistic regression                             Number of obs     =      1,754
                                                Wald chi2(15)     =      70.39
                                                Prob > chi2       =     0.0000
Log pseudolikelihood =  -1164.565               Pseudo R2         =     0.0309

------------------------------------------------------------------------------------------------------
                                       |               Robust
                                  D1_r | Odds Ratio   Std. Err.      z    P>|z|     [95% Conf. Interval]
---------------------------------------+--------------------------------------------------------------
                             condition |
                     DrinkWise Control |   .8655772   .1358061    -0.92   0.358     .6364367    1.177217
                             Text-Only |   .9996556   .1572231    -0.00   0.998     .7344729    1.360583
                      Text + Pictogram |   1.275177   .2009196     1.54   0.123     .9363825    1.736551
                     Text + Photograph |    1.21943   .1893509     1.28   0.201     .8994643    1.653217
                                       |
                       Quota_Age_Group |
                                 30-49 |   .9479119   .1217443    -0.42   0.677      .736961    1.219246
                                 50-69 |   .6759421    .099363    -2.66   0.008      .506738    .9016448
                                       |
                            gender_A2b |
                              Not male |   1.426397   .1629954     3.11   0.002     1.140179    1.784464
                                       |
                              educ_G2b |
                    Tertiary education |   1.264705   .1474497     2.01   0.044      1.00635    1.589385
                                       |
                            parent_G3r |
                                   Yes |   .8816262   .0995502    -1.12   0.265     .7065937    1.100017
                                       |
                          seifa_quin_b |
                               41-100% |    1.02249   .1128456     0.20   0.840     .8236026    1.269407
                                       |
                           NHMRCrisk_b |
               High either STH LTH or Both |   .9298746    .109819    -0.62   0.538     .7377288    1.172066
                                       |
                                 B17_r |
Definitely/Probably drink more than .. |   1.810126   .2045059     5.25   0.000     1.450578    2.258793
                                       |
                                  C1_b |
                                  Wine |   1.051705   .1404893     0.38   0.706      .809447    1.366468
                               Spirits |   .8472784   .1107777    -1.27   0.205     .6557457    1.094755
                                       |
                             base_7day |   .9903649   .0040495    -2.37   0.018     .9824597    .9983337
                                 _cons |   .5355532   .1112995    -3.00   0.003     .3563729    .8048235
------------------------------------------------------------------------------------------------------
Note: _cons estimates baseline odds.

. 
.         **** Omnibus test
.         testparm i.condition

 ( 1)  [D1_r]2.condition = 0
 ( 2)  [D1_r]3.condition = 0
 ( 3)  [D1_r]4.condition = 0
 ( 4)  [D1_r]5.condition = 0

           chi2(  4) =    8.37
         Prob > chi2 =    0.0789

. 
.         **** Adjusted means/proportions
.         margins i.condition, asbal atmeans

Adjusted predictions                            Number of obs     =      1,754
Model VCE    : Robust

Expression   : Pr(D1_r), predict()
at           : condition                     (asbalanced)
               Quota_Age_Group               (asbalanced)
               gender_A2b                    (asbalanced)
               educ_G2b                      (asbalanced)
               parent_G3r                    (asbalanced)
               seifa_quin_b                  (asbalanced)
               NHMRCrisk_b                   (asbalanced)
               B17_r                         (asbalanced)
               C1_b                          (asbalanced)
               base_7day       =    14.12828 (mean)

-------------------------------------------------------------------------------------------------
                                  |            Delta-method
                                  |     Margin   Std. Err.      z    P>|z|     [95% Conf. Interval]
----------------------------------+--------------------------------------------------------------
                        condition |
No Health Warning Label Control |   .3906471   .0286154    13.65   0.000     .3345619    .4467324
                DrinkWise Control |   .3568755   .0272764    13.08   0.000     .3034146    .4103363
                        Text-Only |   .3905651   .0286611    13.63   0.000     .3343904    .4467398
                 Text + Pictogram |   .4497928   .0295664    15.21   0.000     .3918437    .5077419
                Text + Photograph |   .4387568   .0289852    15.14   0.000     .3819468    .4955667
-------------------------------------------------------------------------------------------------
```

```
. di "Intentions to reduce how often and/or how much consumed per occasion in the next month"
Intentions to reduce how often and/or how much consumed per occasion in the next month

.
.         ** Logistic regression
.
.         *** Simple model
.         logistic D2D3_r i.condition ///
>                 $covariates , ///
>                 vce(robust)

Logistic regression                             Number of obs     =      1,754
                                                Wald chi2(15)     =     116.33
                                                Prob > chi2       =     0.0000
Log pseudolikelihood = -1129.8756               Pseudo R2         =     0.0529
```

D2D3_r	Odds Ratio	Robust Std. Err.	z	P>|z|	[95% Conf.	Interval]
condition						
DrinkWise Control	1.051914	.1661957	0.32	0.749	.7717842	1.433721
Text-Only	1.172312	.1844515	1.01	0.312	.8612225	1.595774
Text + Pictogram	1.362083	.2171597	1.94	0.053	.99654	1.861711
Text + Photograph	1.38113	.2179301	2.05	0.041	1.013731	1.881684
Quota_Age_Group						
30-49	.8782249	.1134938	-1.00	0.315	.6817171	1.131377
50-69	.6389361	.092934	-3.08	0.002	.480451	.8497002
gender_A2b						
Not male	1.314823	.152021	2.37	0.018	1.048215	1.64924
educ_G2b						
Tertiary education	1.240768	.1451847	1.84	0.065	.9864837	1.560599
parent_G3r						
Yes	.9041776	.1026885	-0.89	0.375	.7237388	1.129603
seifa_quin_b						
41-100%	1.133027	.1254717	1.13	0.259	.9119642	1.407675
NHMRCrisk_b						
High either STH LTH or Both	.9210647	.1106365	-0.68	0.494	.7278559	1.165561
B17_r						
Definitely/Probably drink more than ..	2.713542	.3188971	8.49	0.000	2.155276	3.416413
C1_b						
Wine	1.072494	.1468947	0.51	0.609	.8199923	1.40275
Spirits	.7950794	.1047179	-1.74	0.082	.6141874	1.029248
base_7day	.9851277	.0036007	-4.10	0.000	.9780956	.9922103
_cons	.8103808	.1678903	-1.01	0.310	.5399354	1.216288

```
Note: _cons estimates baseline odds.

.
.         **** Omnibus test
.         testparm i.condition

 ( 1)  [D2D3_r]2.condition = 0
 ( 2)  [D2D3_r]3.condition = 0
 ( 3)  [D2D3_r]4.condition = 0
 ( 4)  [D2D3_r]5.condition = 0

           chi2(  4) =    6.79
         Prob > chi2 =    0.1475

.
.         **** Adjusted means/proportions
.         margins i.condition, asbal atmeans

Adjusted predictions                            Number of obs     =      1,754
Model VCE    : Robust

Expression   : Pr(D2D3_r), predict()
at           : condition                   (asbalanced)
               Quota_Age_Group             (asbalanced)
               gender_A2b                  (asbalanced)
               educ_G2b                    (asbalanced)
               parent_G3r                  (asbalanced)
               seifa_quin_b                (asbalanced)
               NHMRCrisk_b                 (asbalanced)
               B17_r                       (asbalanced)
               C1_b                        (asbalanced)
               base_7day       =    14.12828 (mean)
```

	Margin	Delta-method Std. Err.	z	P>|z|	[95% Conf.	Interval]
condition						
No Health Warning Label Control	.5117861	.0299684	17.08	0.000	.4530491	.5705232
DrinkWise Control	.5244217	.029654	17.68	0.000	.466301	.5825424
Text-Only	.5513512	.0295305	18.67	0.000	.4934724	.6092299
Text + Pictogram	.5881126	.0297553	19.76	0.000	.5297933	.6464319
Text + Photograph	.5914724	.0294157	20.11	0.000	.5338187	.649126

```
. di "Intentions to avoid drinking alcohol completely in the next month"
Intentions to avoid drinking alcohol completely in the next month

.
.         ** Logistic regression
.
.         *** Simple model
.         logistic D2_D4_4_r i.condition ///
>                 $covariates, ///
>                 vce(robust)

Logistic regression                             Number of obs     =      1,754
                                                Wald chi2(15)     =      70.42
                                                Prob > chi2       =     0.0000
Log pseudolikelihood = -822.27276               Pseudo R2         =     0.0467

------------------------------------------------------------------------------------------------
                                      |               Robust
                            D2_D4_4_r | Odds Ratio   Std. Err.      z    P>|z|     [95% Conf. Interval]
--------------------------------------+---------------------------------------------------------
                            condition |
                     DrinkWise Control |   1.217801   .2528911     0.95   0.343     .8106142    1.829526
                            Text-Only |   1.248006   .2559247     1.08   0.280      .834956    1.865391
                     Text + Pictogram |   1.851557   .3685619     3.09   0.002     1.253433    2.735099
                    Text + Photograph |   1.645378   .3339455     2.45   0.014     1.105366    2.449207
                                      |
                      Quota_Age_Group |
                                30-49 |   .8198903     .13801    -1.18   0.238     .5894883    1.140345
                                50-69 |   .5057728   .1032261    -3.34   0.001     .3390228    .7545398
                                      |
                           gender_A2b |
                             Not male |   .8289013   .1231927    -1.26   0.207     .6194351      1.1092
                                      |
                             educ_G2b |
                   Tertiary education |    1.33647   .2058241     1.88   0.060     .9882554    1.807379
                                      |
                           parent_G3r |
                                  Yes |   1.238765   .1840739     1.44   0.150     .9257737    1.657574
                                      |
                          seifa_quin_b |
                              41-100% |   .6911589   .0925766    -2.76   0.006     .5315751    .8986514
                                      |
                           NHMRCrisk_b |
             High either STH LTH or Both |   .7972699   .1197992    -1.51   0.132     .5938849    1.070307
                                      |
                                B17_r |
Definitely/Probably drink more than .. |   1.865468   .2587117     4.50   0.000     1.421475     2.44814
                                      |
                                 C1_b |
                                 Wine |   .9146437   .1509602    -0.54   0.589     .6618548    1.263983
                              Spirits |   .6868639   .1149102    -2.25   0.025     .4948429    .9533975
                                      |
                            base_7day |    .972305   .0073929    -3.69   0.000     .9579226    .9869033
                                _cons |   .2998694   .0806247    -4.48   0.000     .1770406    .5079153
------------------------------------------------------------------------------------------------
Note: _cons estimates baseline odds.

.
.         **** Omnibus test
.         testparm i.condition

 ( 1)  [D2_D4_4_r]2.condition = 0
 ( 2)  [D2_D4_4_r]3.condition = 0
 ( 3)  [D2_D4_4_r]4.condition = 0
 ( 4)  [D2_D4_4_r]5.condition = 0

           chi2(  4) =   12.70
         Prob > chi2 =    0.0128

.
.         **** Adjusted means/proportions
.         margins i.condition, asbal atmeans

Adjusted predictions                            Number of obs     =      1,754
Model VCE    : Robust

Expression   : Pr(D2_D4_4_r), predict()
at           : condition                      (asbalanced)
               Quota_Age_Group                (asbalanced)
               gender_A2b                     (asbalanced)
               educ_G2b                       (asbalanced)
               parent_G3r                     (asbalanced)
               seifa_quin_b                   (asbalanced)
               NHMRCrisk_b                    (asbalanced)
               B17_r                          (asbalanced)
               C1_b                           (asbalanced)
               base_7day       =    14.12828 (mean)

-----------------------------------------------------------------------------------------
                                 |            Delta-method
                                 |     Margin   Std. Err.      z    P>|z|     [95% Conf. Interval]
---------------------------------+-------------------------------------------------------
                       condition |
No Health Warning Label Control |   .1326708     .019266     6.89   0.000     .0949101    .1704316
               DrinkWise Control |   .1570292    .0205784     7.63   0.000     .1166962    .1973622
                       Text-Only |   .1602997    .0206274     7.77   0.000     .1198707    .2007286
                Text + Pictogram |   .2207122    .0252497     8.74   0.000     .1712237    .2702007
               Text + Photograph |   .2010769    .0244922     8.21   0.000     .1530732    .2490807
-----------------------------------------------------------------------------------------
```

```
. /* FOLLOW-UP */
.
.
. di "Past week alcohol consumption (mean)"
Past week alcohol consumption (mean)

.
.         *** Linear regression
.         nbreg fup_7day i.condition ///
>                 $covariates     ///
>                 if FUP_compl == 1,      ///
>                 robust

Fitting Poisson model:

Iteration 0:   log pseudolikelihood = -24171.376
Iteration 1:   log pseudolikelihood = -11311.426
Iteration 2:   log pseudolikelihood = -5357.4555
Iteration 3:   log pseudolikelihood = -4945.6956
Iteration 4:   log pseudolikelihood = -4929.0752
Iteration 5:   log pseudolikelihood = -4929.0512
Iteration 6:   log pseudolikelihood = -4929.0512

Fitting constant-only model:

Iteration 0:   log pseudolikelihood = -3966.4069
Iteration 1:   log pseudolikelihood = -3965.1749
Iteration 2:   log pseudolikelihood = -3965.1746

Fitting full model:

Iteration 0:   log pseudolikelihood = -3705.7672
Iteration 1:   log pseudolikelihood = -3541.4133
Iteration 2:   log pseudolikelihood = -3517.2905
Iteration 3:   log pseudolikelihood =  -3516.294
Iteration 4:   log pseudolikelihood = -3516.2932
Iteration 5:   log pseudolikelihood = -3516.2932

Negative binomial regression                    Number of obs     =      1,086
                                                Wald chi2(15)     =    1257.64
Dispersion           = mean                     Prob > chi2       =     0.0000
Log pseudolikelihood = -3516.2932               Pseudo R2         =     0.1132

--------------------------------------------------------------------------------------------------------
                                        |               Robust
                               fup_7day |      Coef.   Std. Err.      z    P>|z|     [95% Conf. Interval]
----------------------------------------+---------------------------------------------------------------
                              condition |
                      DrinkWise Control |   .1471645    .069179     2.13   0.033     .0115761    .2827529
                              Text-Only |   .0390657   .0653419     0.60   0.550    -.0890021    .1671335
                       Text + Pictogram |   .0213884    .066804     0.32   0.749    -.1095451    .1523218
                      Text + Photograph |   .0817439   .0730193     1.12   0.263    -.0613712    .2248591
                                        |
                        Quota_Age_Group |
                                  30-49 |   .1312452   .0573594     2.29   0.022     .0188229    .2436675
                                  50-69 |   .1143763   .0529038     2.16   0.031     .0106867    .2180659
                                        |
                             gender_A2b |
                               Not male |  -.1366851   .0493796    -2.77   0.006    -.2334673    -.039903
                                        |
                               educ_G2b |
                     Tertiary education |  -.0488903   .0590248    -0.83   0.407    -.1645768    .0667961
                                        |
                              parent_G3r |
                                    Yes |   .0563037   .0438208     1.28   0.199    -.0295835    .1421909
                                        |
                           seifa_quin_b |
                                41-100% |   .0768751   .0433623     1.77   0.076    -.0081135    .1618637
                                        |
                            NHMRCrisk_b |
               High either STH LTH or Both |   .4037724   .0553767     7.29   0.000      .295236    .5123088
                                        |
                                  B17_r |
Definitely/Probably drink more than should |   .2608546   .0531329     4.91   0.000      .156716    .3649932
                                        |
                                   C1_b |
                                   Wine |  -.0182984   .0532481    -0.34   0.731    -.1226627    .0860659
                                Spirits |  -.0920714   .0565675    -1.63   0.104    -.2029416    .0187988
                                        |
                              base_7day |   .0325991   .0021856    14.92   0.000     .0283155    .0368828
                                  _cons |    1.41218   .0978306    14.43   0.000     1.220435    1.603924
----------------------------------------+---------------------------------------------------------------
                               /lnalpha |  -1.055368   .0796248                     -1.211429   -.8993058
----------------------------------------+---------------------------------------------------------------
                                  alpha |   .3480645   .0277146                      .2977714     .406852
--------------------------------------------------------------------------------------------------------

.
.         **** Omnibus test
.         testparm i.condition

 ( 1)  [fup_7day]2.condition = 0
 ( 2)  [fup_7day]3.condition = 0
 ( 3)  [fup_7day]4.condition = 0
 ( 4)  [fup_7day]5.condition = 0

           chi2(  4) =    5.98
         Prob > chi2 =    0.2008

.
.         **** Adjusted means/proportions
.         margins i.condition, asbal atmeans

Adjusted predictions                            Number of obs     =      1,086
Model VCE    : Robust

Expression   : Predicted number of events, predict()
at           : condition                     (asbalanced)
               Quota_Age_Group               (asbalanced)
               gender_A2b                    (asbalanced)
               educ_G2b                      (asbalanced)
               parent_G3r                    (asbalanced)
               seifa_quin_b                  (asbalanced)
               NHMRCrisk_b                   (asbalanced)
               B17_r                         (asbalanced)
               C1_b                          (asbalanced)
               base_7day       =    14.63444 (mean)

-----------------------------------------------------------------------------------------------
                                 |            Delta-method
                                 |     Margin   Std. Err.      z    P>|z|     [95% Conf. Interval]
---------------------------------+-------------------------------------------------------------
                       condition |
No Health Warning Label Control  |   9.397548   .4924863    19.08   0.000     8.432293     10.3628
              DrinkWise Control  |   10.88748   .5287487    20.59   0.000      9.85115    11.92381
                      Text-Only  |   9.771935   .4615832    21.17   0.000     8.867249    10.67662
               Text + Pictogram  |   9.600711     .46322    20.73   0.000     8.692817    10.50861
              Text + Photograph  |   10.19801    .615355    16.57   0.000     8.991938    11.40408
-----------------------------------------------------------------------------------------------
```

```
.         ** Interaction
.         nbreg fup_7day i.condition##i.RET_sum_b ///
>                 $covariates     ///
>                 if FUP_compl == 1,      ///
>                 robust

Fitting Poisson model:

Iteration 0:   log pseudolikelihood = -23940.676
Iteration 1:   log pseudolikelihood =  -11698.09
Iteration 2:   log pseudolikelihood = -5384.8384
Iteration 3:   log pseudolikelihood = -4905.0167
Iteration 4:   log pseudolikelihood = -4880.5073
Iteration 5:   log pseudolikelihood = -4880.4094
Iteration 6:   log pseudolikelihood = -4880.4094

Fitting constant-only model:

Iteration 0:   log pseudolikelihood = -3966.4069
Iteration 1:   log pseudolikelihood = -3965.1749
Iteration 2:   log pseudolikelihood = -3965.1746

Fitting full model:

Iteration 0:   log pseudolikelihood = -3705.0155
Iteration 1:   log pseudolikelihood = -3539.8851
Iteration 2:   log pseudolikelihood = -3511.6337
Iteration 3:   log pseudolikelihood = -3510.2464
Iteration 4:   log pseudolikelihood = -3510.2448
Iteration 5:   log pseudolikelihood = -3510.2448

Negative binomial regression                    Number of obs     =      1,086
                                                Wald chi2(20)     =    1285.25
Dispersion           = mean                     Prob > chi2       =     0.0000
Log pseudolikelihood = -3510.2448               Pseudo R2         =     0.1147
```

fup_7day	Coef.	Robust Std. Err.	z	P>|z|	[95% Conf.	Interval]
condition						
DrinkWise Control	.2964493	.1301057	2.28	0.023	.0414468	.5514517
Text-Only	.1018786	.1203902	0.85	0.397	-.1340819	.337839
Text + Pictogram	.049798	.1236949	0.40	0.687	-.1926397	.2922356
Text + Photograph	.2640292	.1458601	1.81	0.070	-.0218513	.5499097
RET_sum_b						
High (6-8 exposures)	.0296231	.1125842	0.26	0.792	-.1910379	.2502841
condition#RET_sum_b						
DrinkWise Control#High (6-8 exposures)	-.2400945	.1514684	-1.59	0.113	-.5369672	.0567782
Text-Only#High (6-8 exposures)	-.103637	.1418374	-0.73	0.465	-.3816331	.1743592
Text + Pictogram#High (6-8 exposures)	-.0454069	.1442378	-0.31	0.753	-.3281079	.2372941
Text + Photograph#High (6-8 exposures)	-.2773101	.1655662	-1.67	0.094	-.6018139	.0471937
Quota_Age_Group						
30-49	.1545547	.059482	2.60	0.009	.0379721	.2711373
50-69	.1329145	.0538224	2.47	0.014	.0274245	.2384045
gender_A2b						
Not male	-.1287932	.048061	-2.68	0.007	-.222991	-.0345954
educ_G2b						
Tertiary education	-.0432519	.0548286	-0.79	0.430	-.1507139	.0642102
parent_G3r						
Yes	.0649987	.0430959	1.51	0.131	-.0194676	.149465
seifa_quin_b						
41-100%	.0887692	.0437407	2.03	0.042	.003039	.1744995
NHMRCrisk_b						
High either STH LTH or Both	.4077486	.0543345	7.50	0.000	.3012549	.5142422
B17_r						
Definitely/Probably drink more than should	.2633507	.0520792	5.06	0.000	.1612774	.3654239
C1_b						
Wine	-.0142839	.0530487	-0.27	0.788	-.1182574	.0896895
Spirits	-.0997014	.0557804	-1.79	0.074	-.2090289	.0096261
base_7day	.0327564	.0021503	15.23	0.000	.0285418	.036971
_cons	1.355162	.1106412	12.25	0.000	1.13831	1.572015
/lnalpha	-1.071202	.0770785			-1.222273	-.9201306
alpha	.3425965	.0264068			.2945599	.398467

```
.
.         **** Omnibus test for interaction
.         testparm i.condition#i.RET_sum_b

 ( 1)  [fup_7day]2.condition#1.RET_sum_b = 0
 ( 2)  [fup_7day]3.condition#1.RET_sum_b = 0
 ( 3)  [fup_7day]4.condition#1.RET_sum_b = 0
 ( 4)  [fup_7day]5.condition#1.RET_sum_b = 0

           chi2(  4) =    4.54
         Prob > chi2 =    0.3379
```

```
. di "Intentions to drink less in the next week"
Intentions to drink less in the next week

. 
.         ** Logistic regression
. 
.         *** Simple model
.         logistic FC1_r i.condition ///
>                 $covariates     ///
>                 if FUP_compl == 1,      ///
>                 vce(robust)

Logistic regression                             Number of obs     =      1,086
                                                Wald chi2(15)     =      47.13
                                                Prob > chi2       =     0.0000
Log pseudolikelihood = -726.99946               Pseudo R2         =     0.0335
```

FC1_r	Odds Ratio	Robust Std. Err.	z	P>|z|	[95% Conf.	Interval]
condition						
DrinkWise Control	1.140287	.2296119	0.65	0.514	.7684463	1.692057
Text-Only	1.389915	.2752216	1.66	0.096	.9428425	2.048979
Text + Pictogram	2.004214	.405316	3.44	0.001	1.348354	2.979094
Text + Photograph	1.371456	.2773107	1.56	0.118	.9227147	2.038432
Quota_Age_Group						
30-49	1.072511	.1747434	0.43	0.667	.7793202	1.476003
50-69	.7463149	.1307353	-1.67	0.095	.5294359	1.052037
gender_A2b						
Not male	1.090011	.1577102	0.60	0.551	.8208677	1.4474
educ_G2b						
Tertiary education	1.246161	.193202	1.42	0.156	.9196141	1.688661
parent_G3r						
Yes	1.107307	.1530533	0.74	0.461	.844528	1.451852
seifa_quin_b						
41-100%	.9623781	.1328468	-0.28	0.781	.7342531	1.261379
NHMRCrisk_b						
High either STH LTH or Both	.7914095	.1203112	-1.54	0.124	.58749	1.06611
B17_r						
Definitely/Probably drink more than should	2.146418	.3126398	5.24	0.000	1.61336	2.8556
C1_b						
Wine	.9006297	.1494895	-0.63	0.528	.6505205	1.2469
Spirits	.8478167	.1407535	-0.99	0.320	.6123313	1.173863
base_7day	.9906747	.0046404	-2.00	0.045	.9816214	.9998116
_cons	.5822294	.1618321	-1.95	0.052	.3376763	1.003894

```
Note: _cons estimates baseline odds.

. 
.                 **** Omnibus test
.                 testparm i.condition

 ( 1)  [FC1_r]2.condition = 0
 ( 2)  [FC1_r]3.condition = 0
 ( 3)  [FC1_r]4.condition = 0
 ( 4)  [FC1_r]5.condition = 0

           chi2(  4) =   13.68
         Prob > chi2 =    0.0084

. 
.                 **** Adjusted means/proportions
.                 margins i.condition, asbal atmeans

Adjusted predictions                            Number of obs     =      1,086
Model VCE    : Robust

Expression   : Pr(FC1_r), predict()
at           : condition                     (asbalanced)
               Quota_Age_Group               (asbalanced)
               gender_A2b                    (asbalanced)
               educ_G2b                      (asbalanced)
               parent_G3r                    (asbalanced)
               seifa_quin_b                  (asbalanced)
               NHMRCrisk_b                   (asbalanced)
               B17_r                         (asbalanced)
               C1_b                          (asbalanced)
               base_7day       =    14.63444 (mean)
```

	Margin	Delta-method Std. Err.	z	P>|z|	[95% Conf.	Interval]
condition						
No Health Warning Label Control	.4031709	.0374364	10.77	0.000	.329797	.4765448
DrinkWise Control	.4351203	.0367934	11.83	0.000	.3630066	.5072339
Text-Only	.4842483	.0367419	13.18	0.000	.4122355	.5562611
Text + Pictogram	.5751712	.0369222	15.58	0.000	.502805	.6475373
Text + Photograph	.4809099	.0375783	12.80	0.000	.4072577	.5545621

```
.         ** Interaction
.         logistic FC1_r i.condition##i.RET_sum_b ///
>                 $covariates     ///
>                 if FUP_compl == 1,      ///
>                 vce(robust)

Logistic regression                             Number of obs     =      1,086
                                                Wald chi2(20)     =      50.57
                                                Prob > chi2       =     0.0002
Log pseudolikelihood =  -724.7909               Pseudo R2         =     0.0365

-------------------------------------------------------------------------------------------------------------
                                            |               Robust
                                      FC1_r | Odds Ratio   Std. Err.      z    P>|z|     [95% Conf. Interval]
--------------------------------------------+----------------------------------------------------------------
                                  condition |
                         DrinkWise Control  |   1.100274   .3612677     0.29   0.771      .578115    2.094051
                                 Text-Only  |   1.021692   .3261039     0.07   0.946     .5465541    1.909883
                          Text + Pictogram  |   1.670969   .5535735     1.55   0.121     .8729209    3.198616
                         Text + Photograph  |   1.594518   .5428392     1.37   0.171     .8181734    3.107515
                                            |
                                  RET_sum_b |
                      High (6-8 exposures)  |   .7657826   .2325307    -0.88   0.379     .4223157    1.388589
                                            |
                        condition#RET_sum_b |
    DrinkWise Control#High (6-8 exposures)  |   1.066933   .4452722     0.16   0.877     .4708665    2.417555
            Text-Only#High (6-8 exposures)  |   1.649768   .6730505     1.23   0.220     .7415851    3.670158
     Text + Pictogram#High (6-8 exposures)  |   1.341505   .5608281     0.70   0.482     .5912074       3.044
    Text + Photograph#High (6-8 exposures)  |   .8212505   .3471668    -0.47   0.641     .3586309    1.880631
                                            |
                            Quota_Age_Group |
                                     30-49  |   1.106013   .1843221     0.60   0.545     .7978173    1.533264
                                     50-69  |   .7690427   .1372078    -1.47   0.141     .5421062    1.090979
                                            |
                                 gender_A2b |
                                  Not male  |   1.102648   .1602855     0.67   0.501     .8292828    1.466125
                                            |
                                   educ_G2b |
                        Tertiary education  |   1.246658   .1927953     1.43   0.154     .9206808     1.68805
                                            |
                                 parent_G3r |
                                       Yes  |   1.119913    .155677     0.81   0.415     .8528261    1.470646
                                            |
                               seifa_quin_b |
                                   41-100%  |   .9777271   .1356004    -0.16   0.871      .745015    1.283129
                                            |
                                NHMRCrisk_b |
                 High either STH LTH or Both |  .7973039   .1218391    -1.48   0.138     .5909469     1.07572
                                            |
                                      B17_r |
Definitely/Probably drink more than should  |   2.139864   .3129864     5.20   0.000     1.606517    2.850275
                                            |
                                       C1_b |
                                      Wine  |   .8985205   .1497569    -0.64   0.521     .6481235    1.245656
                                   Spirits  |   .8370721   .1400782    -1.06   0.288      .603004    1.161998
                                            |
                                 base_7day  |    .990815   .0046237    -1.98   0.048     .9817941    .9999188
                                     _cons  |   .6592153   .2182486    -1.26   0.208     .3445221    1.261355
-------------------------------------------------------------------------------------------------------------
Note: _cons estimates baseline odds.

.
.         **** Omnibus test for interaction
.         testparm i.condition#i.RET_sum_b

 ( 1)  [FC1_r]2.condition#1.RET_sum_b = 0
 ( 2)  [FC1_r]3.condition#1.RET_sum_b = 0
 ( 3)  [FC1_r]4.condition#1.RET_sum_b = 0
 ( 4)  [FC1_r]5.condition#1.RET_sum_b = 0

           chi2(  4) =    3.55
         Prob > chi2 =    0.4708
```

```
. di "Intentions to reduce how often and/or how much consumed per occasion in the next month"
Intentions to reduce how often and/or how much consumed per occasion in the next month

.         ** Logistic regression
. 
.         *** Simple model
.         logistic FC2_FC4_3_r i.condition ///
>                 $covariates     ///
>                 if FUP_compl == 1,      ///
>                 vce(robust)

Logistic regression                             Number of obs     =      1,086
                                                Wald chi2(15)     =      43.90
                                                Prob > chi2       =     0.0001
Log pseudolikelihood = -461.01096               Pseudo R2         =     0.0547

--------------------------------------------------------------------------------------------------------------
                                              |               Robust
                                  FC2_FC4_3_r | Odds Ratio   Std. Err.      z    P>|z|     [95% Conf. Interval]
----------------------------------------------+---------------------------------------------------------------
                                    condition |
                            DrinkWise Control |   .6016474   .1809486    -1.69   0.091     .3336866    1.084789
                                    Text-Only |   1.208902    .316214     0.73   0.468     .7240044    2.018556
                             Text + Pictogram |   1.557418   .4021538     1.72   0.086     .9388786    2.583456
                           Text + Photograph  |   1.324193   .3461137     1.07   0.283     .7933531    2.210222
                                              |
                              Quota_Age_Group |
                                        30-49 |   .5609676   .1290159    -2.51   0.012     .3574139    .8804488
                                        50-69 |   .3536873   .0946068    -3.89   0.000     .2093794    .5974548
                                              |
                                   gender_A2b |
                                     Not male |   .6333794   .1294655    -2.23   0.025     .4243016     .945482
                                              |
                                     educ_G2b |
                           Tertiary education |   1.036006   .2266213     0.16   0.872      .674786    1.590591
                                              |
                                   parent_G3r |
                                          Yes |   1.455168   .2903058     1.88   0.060     .9842348    2.151433
                                              |
                                seifa_quin_b |
                                      41-100% |   .8254059   .1507738    -1.05   0.294     .5770079    1.180738
                                              |
                                  NHMRCrisk_b |
                    High either STH LTH or Both |   .7565258    .160694    -1.31   0.189      .498907     1.14717
                                              |
                                        B17_r |
       Definitely/Probably drink more than should |   1.466903   .2852146     1.97   0.049     1.002073    2.147352
                                              |
                                         C1_b |
                                         Wine |   .8959072    .198497    -0.50   0.620     .5803246    1.383105
                                      Spirits |   .6844941   .1543583    -1.68   0.093     .4399645    1.064932
                                              |
                                    base_7day |    .979048   .0107087    -1.94   0.053     .9582827    1.000263
                                        _cons |   .4615474   .1711203    -2.09   0.037     .2231671     .954558
--------------------------------------------------------------------------------------------------------------
Note: _cons estimates baseline odds.

. 
.         **** Omnibus test
.         testparm i.condition

 ( 1)  [FC2_FC4_3_r]2.condition = 0
 ( 2)  [FC2_FC4_3_r]3.condition = 0
 ( 3)  [FC2_FC4_3_r]4.condition = 0
 ( 4)  [FC2_FC4_3_r]5.condition = 0

           chi2(  4) =   12.16
         Prob > chi2 =    0.0162

. 
.         **** Adjusted means/proportions
.         margins i.condition, asbal atmeans

Adjusted predictions                            Number of obs     =      1,086
Model VCE    : Robust

Expression   : Pr(FC2_FC4_3_r), predict()
at           : condition                    (asbalanced)
               Quota_Age_Group              (asbalanced)
               gender_A2b                   (asbalanced)
               educ_G2b                     (asbalanced)
               parent_G3r                   (asbalanced)
               seifa_quin_b                 (asbalanced)
               NHMRCrisk_b                  (asbalanced)
               B17_r                        (asbalanced)
               C1_b                         (asbalanced)
               base_7day       =    14.63444 (mean)

-----------------------------------------------------------------------------------------------
                                |            Delta-method
                                |     Margin   Std. Err.      z    P>|z|     [95% Conf. Interval]
--------------------------------+--------------------------------------------------------------
                      condition |
  No Health Warning Label Control |   .1356194   .0250702     5.41   0.000     .0864826    .1847562
              DrinkWise Control |   .0862549   .0197522     4.37   0.000     .0475414    .1249685
                      Text-Only |   .1594336   .0271037     5.88   0.000     .1063114    .2125558
               Text + Pictogram |   .1963711   .0315547     6.22   0.000      .134525    .2582173
              Text + Photograph |   .1720229   .0284083     6.06   0.000     .1163437    .2277022
-----------------------------------------------------------------------------------------------
```

```
.         ** Interaction
.         logistic FC2_FC4_3_r i.condition##i.RET_sum_b ///
>                 $covariates     ///
>                 if FUP_compl == 1,      ///
>                 vce(robust)

Logistic regression                              Number of obs     =      1,086
                                                 Wald chi2(20)     =      64.25
                                                 Prob > chi2       =     0.0000
Log pseudolikelihood = -451.12103                Pseudo R2         =     0.0750

------------------------------------------------------------------------------------------------------------------
                                              |               Robust
                                  FC2_FC4_3_r | Odds Ratio   Std. Err.      z    P>|z|     [95% Conf. Interval]
----------------------------------------------+-------------------------------------------------------------------
                                    condition |
                            DrinkWise Control |   .4547309   .1958828    -1.83   0.067     .1954724    1.057848
                                    Text-Only |    .967898   .3468549    -0.09   0.927     .4795059    1.953733
                             Text + Pictogram |   1.638614   .5723207     1.41   0.157     .8263734    3.249203
                           Text + Photograph |   .9071232   .3481262    -0.25   0.799     .4275639     1.92456
                                              |
                                    RET_sum_b |
                         High (6-8 exposures) |   .3412313   .1362936    -2.69   0.007     .1559784     .746506
                                              |
                          condition#RET_sum_b |
    DrinkWise Control#High (6-8 exposures)    |   1.832485   1.129702     0.98   0.326     .5473762     6.13472
            Text-Only#High (6-8 exposures)    |   1.632077   .8682715     0.92   0.357     .5753048    4.630025
     Text + Pictogram#High (6-8 exposures)    |   .9638793   .5137987    -0.07   0.945     .3390683    2.740048
    Text + Photograph#High (6-8 exposures)    |   2.254905   1.224655     1.50   0.134     .7777402    6.537655
                                              |
                              Quota_Age_Group |
                                        30-49 |    .679406   .1602298    -1.64   0.101     .4279408    1.078637
                                        50-69 |   .4342727   .1180187    -3.07   0.002     .2549403    .7397525
                                              |
                                   gender_A2b |
                                     Not male |   .6442612   .1332981    -2.12   0.034     .4294848    .9664428
                                              |
                                     educ_G2b |
                           Tertiary education |   1.027638   .2297877     0.12   0.903     .6629866    1.592851
                                              |
                                   parent_G3r |
                                          Yes |   1.523634   .3073251     2.09   0.037     1.026097    2.262416
                                              |
                                  seifa_quin_b |
                                      41-100% |   .8508384   .1562778    -0.88   0.379     .5936119    1.219528
                                              |
                                   NHMRCrisk_b |
                    High either STH LTH or Both |   .7867644   .1690436    -1.12   0.264     .5163643    1.198763
                                              |
                                        B17_r |
Definitely/Probably drink more than should    |   1.377797   .2718345     1.62   0.104     .9359359    2.028263
                                              |
                                         C1_b |
                                         Wine |   .9107458   .2062498    -0.41   0.680     .5842947    1.419588
                                      Spirits |   .6740098    .153466    -1.73   0.083     .4313754    1.053118
                                              |
                                    base_7day |   .9797855   .0109149    -1.83   0.067     .9586245    1.001414
                                        _cons |   .6949271   .2867357    -0.88   0.378     .3095442    1.560112
------------------------------------------------------------------------------------------------------------------
Note: _cons estimates baseline odds.

.
.         **** Omnibus test for interaction
.         testparm i.condition#i.RET_sum_b

 ( 1)  [FC2_FC4_3_r]2.condition#1.RET_sum_b = 0
 ( 2)  [FC2_FC4_3_r]3.condition#1.RET_sum_b = 0
 ( 3)  [FC2_FC4_3_r]4.condition#1.RET_sum_b = 0
 ( 4)  [FC2_FC4_3_r]5.condition#1.RET_sum_b = 0

           chi2(  4) =    3.95
         Prob > chi2 =    0.4131
```

```
. di "Intentions to avoid drinking alcohol completely in the next month"
Intentions to avoid drinking alcohol completely in the next month

.
.         ** Logistic regression
.
.         *** Simple model
.         logistic FC2FC3_r i.condition ///
>                 $covariates     ///
>                 if FUP_compl == 1,      ///
>                 vce(robust)

Logistic regression                             Number of obs     =      1,086
                                                Wald chi2(15)     =      71.72
                                                Prob > chi2       =     0.0000
Log pseudolikelihood = -695.34943               Pseudo R2         =     0.0544

--------------------------------------------------------------------------------------------------------------
                                              |               Robust
                                      FC2FC3_r | Odds Ratio   Std. Err.      z    P>|z|     [95% Conf. Interval]
--------------------------------------------------------------------------------------------------------------
                                     condition |
                              DrinkWise Control  |   1.132096   .2292042     0.61   0.540     .7612877    1.683517
                                     Text-Only  |    1.11087    .225196     0.52   0.604      .746633    1.652795
                              Text + Pictogram  |   1.696494   .3578973     2.51   0.012     1.121967    2.565218
                             Text + Photograph  |   1.254602   .2553595     1.11   0.265     .8418872     1.86964
                                               |
                               Quota_Age_Group |
                                         30-49  |   .7432619   .1236363    -1.78   0.074     .5364763    1.029753
                                         50-69  |    .501721   .0904042    -3.83   0.000     .3524405    .7142311
                                               |
                                     gender_A2b |
                                      Not male  |   1.083193   .1618641     0.53   0.593     .8081811    1.451787
                                               |
                                      educ_G2b |
                             Tertirary education |   1.419474   .2246622     2.21   0.027     1.040894    1.935745
                                               |
                                     parent_G3r |
                                           Yes  |   1.128114   .1594484     0.85   0.394     .8551526    1.488204
                                               |
                                   seifa_quin_b |
                                       41-100%  |   1.355826   .1913045     2.16   0.031     1.028256    1.787751
                                               |
                                    NHMRCrisk_b |
                       High either STH LTH or Both |   .7643387   .1199535    -1.71   0.087     .5619533    1.039612
                                               |
                                         B17_r |
       Definitely/Probably drink more than should |    2.61036   .3946068     6.35   0.000     1.940997    3.510558
                                               |
                                          C1_b |
                                          Wine  |    .916007   .1584079    -0.51   0.612     .6526764    1.285582
                                       Spirits  |   .7571115   .1283442    -1.64   0.101     .5430823     1.05549
                                               |
                                     base_7day |    .993473   .0048104    -1.35   0.176     .9840894    1.002946
                                         _cons |   .8038174   .2244426    -0.78   0.434     .4650334    1.389411
--------------------------------------------------------------------------------------------------------------
Note: _cons estimates baseline odds.

.
.         **** Omnibus test
.         testparm i.condition

 ( 1)  [FC2FC3_r]2.condition = 0
 ( 2)  [FC2FC3_r]3.condition = 0
 ( 3)  [FC2FC3_r]4.condition = 0
 ( 4)  [FC2FC3_r]5.condition = 0

           chi2(  4) =    7.24
         Prob > chi2 =    0.1238

.
.         **** Adjusted means/proportions
.         margins i.condition, asbal atmeans

Adjusted predictions                            Number of obs     =      1,086
Model VCE    : Robust

Expression   : Pr(FC2FC3_r), predict()
at           : condition                     (asbalanced)
               Quota_Age_Group               (asbalanced)
               gender_A2b                    (asbalanced)
               educ_G2b                      (asbalanced)
               parent_G3r                    (asbalanced)
               seifa_quin_b                  (asbalanced)
               NHMRCrisk_b                   (asbalanced)
               B17_r                         (asbalanced)
               C1_b                          (asbalanced)
               base_7day       =    14.63444 (mean)

-----------------------------------------------------------------------------------------------
                                 |            Delta-method
                                 |     Margin   Std. Err.      z    P>|z|     [95% Conf. Interval]
-----------------------------------------------------------------------------------------------
                       condition |
  No Health Warning Label Control  |   .5019845   .0387537    12.95   0.000     .4260287    .5779403
               DrinkWise Control  |   .5329543   .0373628    14.26   0.000     .4597246    .6061839
                       Text-Only  |   .5282402   .0383481    13.77   0.000     .4530794     .603401
                Text + Pictogram  |   .6309982   .0375659    16.80   0.000     .5573704     .704626
               Text + Photograph  |    .558421   .0380759    14.67   0.000     .4837937    .6330483
-----------------------------------------------------------------------------------------------
```

```
.         ** Interaction
.
.         *** Linear regression
.         logistic FC2FC3_r i.condition##i.RET_sum_b ///
>                $covariates     ///
>                if FUP_compl == 1,      ///
>                vce(robust)

Logistic regression                               Number of obs     =      1,086
                                                  Wald chi2(20)     =      76.89
                                                  Prob > chi2       =     0.0000
Log pseudolikelihood =  -692.5155                 Pseudo R2         =     0.0582

--------------------------------------------------------------------------------------------------------------
                                            |               Robust
                                   FC2FC3_r | Odds Ratio   Std. Err.      z    P>|z|     [95% Conf. Interval]
--------------------------------------------+-----------------------------------------------------------------
                                  condition |
                          DrinkWise Control |   1.204659   .4133475     0.54   0.587     .6148884    2.360108
                                  Text-Only |   .9415769   .3121331    -0.18   0.856     .4916796     1.80314
                           Text + Pictogram |   1.631854   .5926247     1.35   0.178      .800864    3.325094
                          Text + Photograph |   1.905587   .6808876     1.80   0.071     .9459861    3.838598
                                            |
                                  RET_sum_b |
                       High (6-8 exposures) |   .9066395   .2786086    -0.32   0.750     .4964346    1.655798
                                            |
                        condition#RET_sum_b |
    DrinkWise Control#High (6-8 exposures) |   .9097683   .3872532    -0.22   0.824     .3950092     2.09534
            Text-Only#High (6-8 exposures) |   1.305292   .5473349     0.64   0.525     .5738286    2.969159
     Text + Pictogram#High (6-8 exposures) |    1.06409   .4731698     0.14   0.889     .4451171    2.543799
    Text + Photograph#High (6-8 exposures) |   .5540615   .2395809    -1.37   0.172     .2374057    1.293078
                                            |
                            Quota_Age_Group |
                                      30-49 |   .7681295   .1307814    -1.55   0.121     .5501857    1.072407
                                      50-69 |   .5172107   .0950231    -3.59   0.000     .3608136    .7413992
                                            |
                                 gender_A2b |
                                   Not male |    1.09703    .165056     0.62   0.538     .8168626    1.473289
                                            |
                                   educ_G2b |
                         Tertiary education |   1.418635   .2248042     2.21   0.027     1.039884    1.935336
                                            |
                                 parent_G3r |
                                        Yes |   1.149127   .1628782     0.98   0.327     .8703984    1.517114
                                            |
                               seifa_quin_b |
                                    41-100% |   1.392445   .1972106     2.34   0.019     1.054929    1.837947
                                            |
                                NHMRCrisk_b |
                 High either STH LTH or Both |   .7729164   .1218156    -1.63   0.102     .5675168    1.052656
                                            |
                                      B17_r |
Definitely/Probably drink more than should |   2.609535    .395921     6.32   0.000     1.938287    3.513243
                                            |
                                       C1_b |
                                       Wine |   .9114231   .1582957    -0.53   0.593     .6484606    1.281022
                                    Spirits |   .7423208   .1263366    -1.75   0.080     .5317708    1.036236
                                            |
                                  base_7day |   .9936328   .0047777    -1.33   0.184     .9843127    1.003041
                                      _cons |   .8109393   .2736492    -0.62   0.535     .4185553    1.571173
--------------------------------------------------------------------------------------------------------------
Note: _cons estimates baseline odds.

.
.         **** Omnibus test for interaction
.         testparm i.condition#i.RET_sum_b

 ( 1)  [FC2FC3_r]2.condition#1.RET_sum_b = 0
 ( 2)  [FC2FC3_r]3.condition#1.RET_sum_b = 0
 ( 3)  [FC2FC3_r]4.condition#1.RET_sum_b = 0
 ( 4)  [FC2FC3_r]5.condition#1.RET_sum_b = 0

           chi2(  4) =    4.44
         Prob > chi2 =    0.3500
```

```
. di "Readiness to change (mean)"
Readiness to change (mean)

.
.
.         ** Logistic regression
.
.         *** Linear regression
.         regress FC7 i.condition ///
>                 $covariates     ///
>                 if FUP_compl == 1,      ///
>                 vce(hc3)

Linear regression                               Number of obs     =      1,086
                                                F(15, 1070)       =       3.69
                                                Prob > F          =     0.0000
                                                R-squared         =     0.0454
                                                Root MSE          =     2.7018

--------------------------------------------------------------------------------------------------------------
                                             |             Robust HC3
                                         FC7 |      Coef.   Std. Err.      t    P>|t|     [95% Conf. Interval]
---------------------------------------------+----------------------------------------------------------------
                                   condition |
                           DrinkWise Control |   .0771021   .2720359     0.28   0.777    -.4566823    .6108864
                                   Text-Only |   .0517547   .2676778     0.19   0.847    -.4734783    .5769876
                            Text + Pictogram |   .4783239   .2735402     1.75   0.081    -.0584122     1.01506
                           Text + Photograph |   .3692183   .2698701     1.37   0.172    -.1603163    .8987529
                                             |
                             Quota_Age_Group |
                                       30-49 |  -.2407681   .2218747    -1.09   0.278     -.676127    .1945908
                                       50-69 |  -.5395311   .2420756    -2.23   0.026    -1.014528   -.0645343
                                             |
                                  gender_A2b |
                                    Not male |  -.2189946   .1918297    -1.14   0.254    -.5953997    .1574105
                                             |
                                    educ_G2b |
                          Tertirary education |  -.1346163   .2110999    -0.64   0.524    -.5488331    .2796005
                                             |
                                  parent_G3r |
                                         Yes |   .4266602   .1919189     2.22   0.026     .0500801    .8032403
                                             |
                                seifa_quin_b |
                                     41-100% |   .2307057   .1900172     1.21   0.225    -.1421429    .6035544
                                             |
                                 NHMRCrisk_b |
                   High either STH LTH or Both |  -.2599072   .2059757    -1.26   0.207    -.6640694     .144255
                                             |
                                       B17_r |
  Definitely/Probably drink more than should |   .8132415   .1886566     4.31   0.000     .4430626     1.18342
                                             |
                                        C1_b |
                                        Wine |  -.0321384   .2205949    -0.15   0.884    -.4649859    .4007092
                                     Spirits |  -.6955017   .2171456    -3.20   0.001    -1.121581   -.2694223
                                             |
                                   base_7day |  -.0170599   .0061229    -2.79   0.005    -.0290742   -.0050456
                                       _cons |    4.70192   .3745832    12.55   0.000      3.96692    5.436921
--------------------------------------------------------------------------------------------------------------

.
.         **** Omnibus test
.         testparm i.condition

 ( 1)  2.condition = 0
 ( 2)  3.condition = 0
 ( 3)  4.condition = 0
 ( 4)  5.condition = 0

       F(  4,  1070) =    1.32
            Prob > F =    0.2610
```

```
.         **** Adjusted means/proportions
.         margins i.condition, asbal atmeans

Adjusted predictions                            Number of obs     =      1,086
Model VCE    : Robust HC3

Expression   : Linear prediction, predict()
at           : condition                     (asbalanced)
               Quota_Age_Group               (asbalanced)
               gender_A2b                    (asbalanced)
               educ_G2b                      (asbalanced)
               parent_G3r                    (asbalanced)
               seifa_quin_b                  (asbalanced)
               NHMRCrisk_b                   (asbalanced)
               B17_r                         (asbalanced)
               C1_b                          (asbalanced)
               base_7day       =    14.63444 (mean)
```

	Margin	Delta-method Std. Err.	t	P>|t|	[95% Conf.	Interval]
condition						
No Health Warning Label Control	4.378156	.2162186	20.25	0.000	3.953896	4.802417
DrinkWise Control	4.455258	.2059058	21.64	0.000	4.051233	4.859283
Text-Only	4.429911	.1961685	22.58	0.000	4.044992	4.81483
Text + Pictogram	4.85648	.199283	24.37	0.000	4.46545	5.24751
Text + Photograph	4.747375	.2025278	23.44	0.000	4.349978	5.144771

```
.
.         ** Interaction
.
.         *** Linear regression
.         regress FC7 i.condition##i.RET_sum_b ///
>                 $covariates     ///
>                 if FUP_compl == 1,      ///
>                 vce(hc3)

Linear regression                               Number of obs     =      1,086
                                                F(20, 1065)       =       3.04
                                                Prob > F          =     0.0000
                                                R-squared         =     0.0520
                                                Root MSE          =     2.6987
```

FC7	Coef.	Robust HC3 Std. Err.	t	P>|t|	[95% Conf.	Interval]
condition						
DrinkWise Control	-.6438	.4556198	-1.41	0.158	-1.537814	.2502145
Text-Only	-.6341069	.4444678	-1.43	0.154	-1.506239	.2380251
Text + Pictogram	.042286	.4729967	0.09	0.929	-.8858253	.9703973
Text + Photograph	-.0670403	.4369708	-0.15	0.878	-.9244618	.7903811
RET_sum_b						
High (6-8 exposures)	-.9829455	.4211391	-2.33	0.020	-1.809302	-.156589
condition#RET_sum_b						
DrinkWise Control#High (6-8 exposures)	1.156355	.5704784	2.03	0.043	.0369654	2.275744
Text-Only#High (6-8 exposures)	1.108564	.5568409	1.99	0.047	.0159342	2.201194
Text + Pictogram#High (6-8 exposures)	.7068364	.5770757	1.22	0.221	-.425498	1.839171
Text + Photograph#High (6-8 exposures)	.7300755	.5532219	1.32	0.187	-.3554533	1.815604
Quota_Age_Group						
30-49	-.1709335	.225056	-0.76	0.448	-.6125371	.2706701
50-69	-.4567283	.2435619	-1.88	0.061	-.934644	.0211874
gender_A2b						
Not male	-.2163845	.1931698	-1.12	0.263	-.5954211	.1626522
educ_G2b						
Tertiary education	-.1368404	.2099821	-0.65	0.515	-.5488659	.2751851
parent_G3r						
Yes	.4490594	.1925569	2.33	0.020	.0712254	.8268933
seifa_quin_b						
41-100%	.2329219	.1906278	1.22	0.222	-.1411267	.6069706
NHMRCrisk_b						
High either STH LTH or Both	-.2549448	.2064049	-1.24	0.217	-.6599514	.1500617
B17_r						
Definitely/Probably drink more than should	.7860334	.1903953	4.13	0.000	.412441	1.159626
C1_b						
Wine	-.0251735	.2210388	-0.11	0.909	-.4588946	.4085476
Spirits	-.6928766	.2179444	-3.18	0.002	-1.120526	-.2652274
base_7day	-.0166771	.0062767	-2.66	0.008	-.0289933	-.004361
_cons	5.260173	.456633	11.52	0.000	4.36417	6.156175

```
.
.         **** Omnibus test for interaction
.         testparm i.condition#i.RET_sum_b

 ( 1)  2.condition#1.RET_sum_b = 0
 ( 2)  3.condition#1.RET_sum_b = 0
 ( 3)  4.condition#1.RET_sum_b = 0
 ( 4)  5.condition#1.RET_sum_b = 0

       F(  4,  1065) =    1.32
            Prob > F =    0.2607
```

```
. di "Frequency of thinking about alcohol-related health risks in the past week"
Frequency of thinking about alcohol-related health risks in the past week

.
.
.         ** Logistic regression
.
.         *** Simple model
.         logistic FD1_r i.condition ///
>                 $covariates     ///
>                 if FUP_compl == 1,      ///
>                 vce(robust)

Logistic regression                             Number of obs     =      1,086
                                                Wald chi2(15)     =      45.36
                                                Prob > chi2       =     0.0001
Log pseudolikelihood = -660.86017               Pseudo R2         =     0.0371
```

FD1_r	Odds Ratio	Robust Std. Err.	z	P>|z|	[95% Conf.	Interval]
condition						
DrinkWise Control	1.149811	.2465414	0.65	0.515	.7552876	1.750412
Text-Only	1.128857	.2361104	0.58	0.562	.7492084	1.700886
Text + Pictogram	1.535724	.3284522	2.01	0.045	1.009864	2.335412
Text + Photograph	1.055024	.2242013	0.25	0.801	.6956247	1.600109
Quota_Age_Group						
30-49	.8213734	.1411841	-1.14	0.252	.5864479	1.150408
50-69	.4207596	.0846844	-4.30	0.000	.2836067	.6242401
gender_A2b						
Not male	.8224429	.1268423	-1.27	0.205	.6078944	1.112714
educ_G2b						
Tertiary education	1.356151	.2341906	1.76	0.078	.9667545	1.902393
parent_G3r						
Yes	.9845199	.1478628	-0.10	0.917	.7334736	1.321492
seifa_quin_b						
41-100%	.8251639	.1203655	-1.32	0.188	.6199785	1.098257
NHMRCrisk_b						
High either STH LTH or Both	.6667203	.109298	-2.47	0.013	.4835071	.9193574
B17_r						
Definitely/Probably drink more than should	1.580945	.2388602	3.03	0.002	1.17574	2.1258
C1_b						
Wine	.8959849	.154046	-0.64	0.523	.6396674	1.25501
Spirits	.6509758	.1164379	-2.40	0.016	.4584725	.9243074
base_7day	1.003809	.0047943	0.80	0.426	.9944567	1.01325
_cons	.6104058	.1800977	-1.67	0.094	.3423538	1.088334

```
Note: _cons estimates baseline odds.

.
.         **** Omnibus test
.         testparm i.condition

 ( 1)  [FD1_r]2.condition = 0
 ( 2)  [FD1_r]3.condition = 0
 ( 3)  [FD1_r]4.condition = 0
 ( 4)  [FD1_r]5.condition = 0

           chi2(  4) =    4.93
         Prob > chi2 =    0.2941
```

```
.
.         **** Adjusted means/proportions
.         margins i.condition, asbal atmeans

Adjusted predictions                            Number of obs     =      1,086
Model VCE    : Robust

Expression   : Pr(FD1_r), predict()
at           : condition                     (asbalanced)
               Quota_Age_Group               (asbalanced)
               gender_A2b                    (asbalanced)
               educ_G2b                      (asbalanced)
               parent_G3r                    (asbalanced)
               seifa_quin_b                  (asbalanced)
               NHMRCrisk_b                   (asbalanced)
               B17_r                         (asbalanced)
               C1_b                          (asbalanced)
               base_7day       =    14.63444 (mean)
```

	Margin	Delta-method Std. Err.	z	P>|z|	[95% Conf.	Interval]
condition						
No Health Warning Label Control	.269959	.0329579	8.19	0.000	.2053628	.3345552
DrinkWise Control	.2983362	.0341982	8.72	0.000	.2313089	.3653635
Text-Only	.2945006	.0333637	8.83	0.000	.2291089	.3598923
Text + Pictogram	.3621999	.037078	9.77	0.000	.2895283	.4348715
Text + Photograph	.2806445	.0325303	8.63	0.000	.2168863	.3444027

```
.
.         ** Interaction
.
.         *** Simple model
.         logistic FD1_r i.condition##i.RET_sum_b ///
>                 $covariates     ///
>                 if FUP_compl == 1,      ///
>                 vce(robust)

Logistic regression                             Number of obs     =      1,086
                                                Wald chi2(20)     =      46.34
                                                Prob > chi2       =     0.0007
Log pseudolikelihood = -660.03462               Pseudo R2         =     0.0383
```

FD1_r	Odds Ratio	Robust Std. Err.	z	P>|z|	[95% Conf.	Interval]
condition						
DrinkWise Control	.835694	.2873791	-0.52	0.602	.4259273	1.63968
Text-Only	.9870374	.3153714	-0.04	0.967	.5276714	1.846306
Text + Pictogram	1.326389	.4482503	0.84	0.403	.6839264	2.572365
Text + Photograph	.8199839	.2842277	-0.57	0.567	.4156778	1.617536
RET_sum_b						
High (6-8 exposures)	.7293837	.2263035	-1.02	0.309	.3970603	1.339848
condition#RET_sum_b						
DrinkWise Control#High (6-8 exposures)	1.6826	.7408178	1.18	0.237	.7099254	3.987946
Text-Only#High (6-8 exposures)	1.256345	.5312301	0.54	0.589	.5485155	2.877591
Text + Pictogram#High (6-8 exposures)	1.283633	.5596323	0.57	0.567	.5461829	3.016781
Text + Photograph#High (6-8 exposures)	1.51185	.6664357	0.94	0.348	.6372243	3.586949
Quota_Age_Group						
30-49	.832854	.1470975	-1.04	0.300	.5891568	1.177354
50-69	.4283216	.0877326	-4.14	0.000	.2866945	.6399125
gender_A2b						
Not male	.8179933	.1263708	-1.30	0.193	.6042945	1.107263
educ_G2b						
Tertiary education	1.353949	.2347529	1.75	0.081	.9638684	1.901897
parent_G3r						
Yes	.9901439	.1483595	-0.07	0.947	.7381718	1.328126
seifa_quin_b						
41-100%	.8197109	.1199629	-1.36	0.174	.6153033	1.092024
NHMRCrisk_b						
High either STH LTH or Both	.6649009	.1091284	-2.49	0.013	.4820049	.9171965
B17_r						
Definitely/Probably drink more than should	1.569232	.2377783	2.97	0.003	1.166027	2.111863
C1_b						
Wine	.8993054	.154856	-0.62	0.538	.6417036	1.260317
Spirits	.6569302	.1175441	-2.35	0.019	.4626092	.9328767
base_7day	1.003911	.0048336	0.81	0.418	.9944815	1.013429
_cons	.7349339	.2510318	-0.90	0.367	.3762729	1.435468

```
Note: _cons estimates baseline odds.

.
.         **** Omnibus test for interaction
.         testparm i.condition#i.RET_sum_b

 ( 1)  [FD1_r]2.condition#1.RET_sum_b = 0
 ( 2)  [FD1_r]3.condition#1.RET_sum_b = 0
 ( 3)  [FD1_r]4.condition#1.RET_sum_b = 0
 ( 4)  [FD1_r]5.condition#1.RET_sum_b = 0

           chi2(  4) =    1.63
         Prob > chi2 =    0.8038
```

```
. di "Increase your risk of cancer"
Increase your risk of cancer

.
.         ** Logistic regression
.
.         *** Simple model
.         logistic FD2_FD6_1_b i.condition ///
>                 $covariates     ///
>                 if FUP_compl == 1,      ///
>                 vce(robust)

Logistic regression                             Number of obs     =      1,086
                                                Wald chi2(15)     =      36.11
                                                Prob > chi2       =     0.0017
Log pseudolikelihood = -676.75525               Pseudo R2         =     0.0286

--------------------------------------------------------------------------------------------------------------
                                             |               Robust
                                 FD2_FD6_1_b | Odds Ratio   Std. Err.      z    P>|z|     [95% Conf. Interval]
---------------------------------------------+----------------------------------------------------------------
                                   condition |
                           DrinkWise Control |   .8190231   .1640185    -1.00   0.319     .5531381    1.212715
                                   Text-Only |   1.268224   .2633384     1.14   0.252     .8442081    1.905208
                            Text + Pictogram |   1.325797    .281197     1.33   0.184     .8748634    2.009158
                           Text + Photograph |   1.372612   .2835643     1.53   0.125     .9155882    2.057762
                                             |
                             Quota_Age_Group |
                                       30-49 |   .8988074   .1521756    -0.63   0.529     .6449872    1.252513
                                       50-69 |   .6707413   .1214409    -2.21   0.027     .4703714    .9564652
                                             |
                                  gender_A2b |
                                    Not male |   1.025124   .1548021     0.16   0.869     .7624972    1.378208
                                             |
                                    educ_G2b |
                          Tertiary education |    .905637   .1480209    -0.61   0.544      .657401    1.247607
                                             |
                                  parent_G3r |
                                         Yes |   1.120137   .1602703     0.79   0.428     .8462146     1.48273
                                             |
                                seifa_quin_b |
                                     41-100% |   1.313772   .1876771     1.91   0.056     .9929397     1.73827
                                             |
                                 NHMRCrisk_b |
                     High either STH LTH or Both |   .8109323   .1278597    -1.33   0.184     .5953545    1.104571
                                             |
                                       B17_r |
Definitely/Probably drink more than should |   1.846489   .2836048     3.99   0.000       1.3665    2.495077
                                             |
                                        C1_b |
                                        Wine |   1.125629   .1962003     0.68   0.497     .7998867    1.584025
                                     Spirits |   .8051101   .1365393    -1.28   0.201       .57743    1.122564
                                             |
                                   base_7day |   .9957386   .0049611    -0.86   0.391     .9860624     1.00551
                                       _cons |   1.537102   .4425806     1.49   0.135     .8742081    2.702656
--------------------------------------------------------------------------------------------------------------
Note: _cons estimates baseline odds.

.
.         **** Omnibus test
.         testparm i.condition

 ( 1)  [FD2_FD6_1_b]2.condition = 0
 ( 2)  [FD2_FD6_1_b]3.condition = 0
 ( 3)  [FD2_FD6_1_b]4.condition = 0
 ( 4)  [FD2_FD6_1_b]5.condition = 0

           chi2(  4) =    9.59
         Prob > chi2 =    0.0480
```

```
.         **** Adjusted means/proportions
.         margins i.condition, asbal atmeans

Adjusted predictions                            Number of obs     =      1,086
Model VCE    : Robust

Expression   : Pr(FD2_FD6_1_b), predict()
at           : condition                     (asbalanced)
               Quota_Age_Group               (asbalanced)
               gender_A2b                    (asbalanced)
               educ_G2b                      (asbalanced)
               parent_G3r                    (asbalanced)
               seifa_quin_b                  (asbalanced)
               NHMRCrisk_b                   (asbalanced)
               B17_r                         (asbalanced)
               C1_b                          (asbalanced)
               base_7day       =    14.63444 (mean)

------------------------------------------------------------------------------------------------
                                 |            Delta-method
                                 |     Margin   Std. Err.      z    P>|z|     [95% Conf. Interval]
---------------------------------+--------------------------------------------------------------
                       condition |
No Health Warning Label Control  |    .6280331   .0365047    17.20   0.000     .5564852    .6995809
              DrinkWise Control  |    .5803341   .0363684    15.96   0.000     .5090533    .6516149
                      Text-Only  |    .6816588   .0353972    19.26   0.000     .6122815    .7510361
               Text + Pictogram  |    .6912142   .0345163    20.03   0.000     .6235635    .7588649
              Text + Photograph  |    .6985712   .0333907    20.92   0.000     .6331265    .7640158
------------------------------------------------------------------------------------------------

.
.         ** Interaction
.
.         *** Simple model
.         logistic FD2_FD6_1_b i.condition##i.RET_sum_b ///
>                 $covariates ///
>                 if FUP_compl == 1,      ///
>                 vce(robust)

Logistic regression                             Number of obs     =      1,086
                                                Wald chi2(20)     =      39.84
                                                Prob > chi2       =     0.0052
Log pseudolikelihood =  -674.7703               Pseudo R2         =     0.0314

----------------------------------------------------------------------------------------------------------
                                          |               Robust
                              FD2_FD6_1_b | Odds Ratio   Std. Err.      z    P>|z|     [95% Conf. Interval]
------------------------------------------+---------------------------------------------------------------
                                condition |
                       DrinkWise Control  |   .7773572   .2499048    -0.78   0.433     .4139772    1.459704
                               Text-Only  |   1.414924   .4702788     1.04   0.296      .737597    2.714234
                        Text + Pictogram  |   1.451104   .4935498     1.09   0.274     .7450542    2.826242
                       Text + Photograph  |   1.383864   .4663656     0.96   0.335     .7148851    2.678862
                                          |
                                RET_sum_b |
                     High (6-8 exposures) |   1.371437   .4082572     1.06   0.289     .7652181    2.457912
                                          |
                      condition#RET_sum_b |
    DrinkWise Control#High (6-8 exposures)|   1.081074   .4450482     0.19   0.850     .4824359    2.422543
            Text-Only#High (6-8 exposures)|   .8412335   .3596249    -0.40   0.686     .3639405    1.944477
     Text + Pictogram#High (6-8 exposures)|   .8637125   .3734642    -0.34   0.735     .3700962     2.01569
    Text + Photograph#High (6-8 exposures)|   .9638584   .4096658    -0.09   0.931      .419015    2.217159
                                          |
                          Quota_Age_Group |
                                   30-49  |   .8430659   .1458617    -0.99   0.324     .6006096    1.183398
                                   50-69  |   .6287536   .1167413    -2.50   0.012     .4369553    .9047403
                                          |
                               gender_A2b |
                                Not male  |   1.020364   .1544218     0.13   0.894     .7584625      1.3727
                                          |
                                 educ_G2b |
                      Tertiary education  |   .9074402   .1492653    -0.59   0.555     .6573602    1.252658
                                          |
                               parent_G3r |
                                     Yes  |   1.105949   .1594612     0.70   0.485     .8336905     1.46712
                                          |
                             seifa_quin_b |
                                 41-100%  |   1.293283   .1852303     1.80   0.073     .9767435    1.712407
                                          |
                              NHMRCrisk_b |
                High either STH LTH or Both|    .798428   .1261756    -1.42   0.154     .5857609    1.088306
                                          |
                                    B17_r |
Definitely/Probably drink more than should|   1.865281   .2874878     4.04   0.000     1.378961     2.52311
                                          |
                                     C1_b |
                                    Wine  |   1.112926   .1948765     0.61   0.541     .7896207    1.568607
                                 Spirits  |    .811917   .1384679    -1.22   0.222     .5812247    1.134173
                                          |
                                base_7day |   .9956954   .0049046    -0.88   0.381     .9861288    1.005355
                                    _cons |   1.342683   .4469504     0.89   0.376     .6992405    2.578222
----------------------------------------------------------------------------------------------------------
Note: _cons estimates baseline odds.

.
.         **** Omnibus test for interaction
.         testparm i.condition#i.RET_sum_b

 ( 1)  [FD2_FD6_1_b]2.condition#1.RET_sum_b = 0
 ( 2)  [FD2_FD6_1_b]3.condition#1.RET_sum_b = 0
 ( 3)  [FD2_FD6_1_b]4.condition#1.RET_sum_b = 0
 ( 4)  [FD2_FD6_1_b]5.condition#1.RET_sum_b = 0

           chi2(  4) =    0.48
         Prob > chi2 =    0.9755
```

```
. di "Increase your risk of liver damage"
Increase your risk of liver damage

. 
.         ** Logistic regression
. 
.         *** Simple model
.         logistic FD2_FD6_2_b i.condition ///
>                 $covariates     ///
>                 if FUP_compl == 1,      ///
>                 vce(robust)

Logistic regression                             Number of obs     =      1,086
                                                Wald chi2(15)     =      40.85
                                                Prob > chi2       =     0.0003
Log pseudolikelihood =   -371.297               Pseudo R2         =     0.0424

--------------------------------------------------------------------------------------------------------------
                                             |               Robust
                                 FD2_FD6_2_b | Odds Ratio   Std. Err.      z    P>|z|     [95% Conf. Interval]
---------------------------------------------+----------------------------------------------------------------
                                   condition |
                           DrinkWise Control |   .9576438   .2897359    -0.14   0.886      .529264    1.732749
                                   Text-Only |   .8428974   .2476052    -0.58   0.561     .4739474    1.499061
                            Text + Pictogram |   1.189839    .379798     0.54   0.586     .6364782    2.224296
                           Text + Photograph |   1.340418   .4176651     0.94   0.347     .7277996    2.468703
                                             |
                             Quota_Age_Group |
                                       30-49 |   1.271168   .3209405     0.95   0.342     .7749865    2.085027
                                       50-69 |   1.045276   .2922195     0.16   0.874     .6043208    1.807985
                                             |
                                  gender_A2b |
                                    Not male |   1.554029   .3735564     1.83   0.067     .9701701     2.48926
                                             |
                                    educ_G2b |
                          Tertiary education |   .7948254   .2075255    -0.88   0.379     .4764602    1.325919
                                             |
                                  parent_G3r |
                                         Yes |   1.264604   .2767098     1.07   0.283     .8235727    1.941812
                                             |
                                seifa_quin_b |
                                     41-100% |   1.089439   .2327902     0.40   0.688     .7166699    1.656101
                                             |
                                  NHMRCrisk_b |
                  High either STH LTH or Both |   .8951885    .212071    -0.47   0.640     .5626833     1.42418
                                             |
                                       B17_r |
     Definitely/Probably drink more than should |   1.413626   .3247129     1.51   0.132     .9011801    2.217467
                                             |
                                        C1_b |
                                        Wine |    2.17935   .6001736     2.83   0.005     1.270322     3.73887
                                     Spirits |   1.569281   .3923698     1.80   0.072     .9613318    2.561698
                                             |
                                   base_7day |   1.008921   .0082597     1.08   0.278     .9928611     1.02524
                                       _cons |   3.250595   1.505686     2.54   0.011     1.311244    8.058277
--------------------------------------------------------------------------------------------------------------
Note: _cons estimates baseline odds.

. 
.         **** Omnibus test
.         testparm i.condition

 ( 1)  [FD2_FD6_2_b]2.condition = 0
 ( 2)  [FD2_FD6_2_b]3.condition = 0
 ( 3)  [FD2_FD6_2_b]4.condition = 0
 ( 4)  [FD2_FD6_2_b]5.condition = 0

           chi2(  4) =    2.90
         Prob > chi2 =    0.5742
```

```
.         **** Adjusted means/proportions
.         margins i.condition, asbal atmeans

Adjusted predictions                             Number of obs     =      1,086
Model VCE    : Robust

Expression   : Pr(FD2_FD6_2_b), predict()
at           : condition                      (asbalanced)
               Quota_Age_Group                (asbalanced)
               gender_A2b                     (asbalanced)
               educ_G2b                       (asbalanced)
               parent_G3r                     (asbalanced)
               seifa_quin_b                   (asbalanced)
               NHMRCrisk_b                    (asbalanced)
               B17_r                          (asbalanced)
               C1_b                           (asbalanced)
               base_7day       =    14.63444 (mean)

-------------------------------------------------------------------------------------------------
                                 |            Delta-method
                                 |     Margin   Std. Err.      z    P>|z|     [95% Conf. Interval]
---------------------------------+---------------------------------------------------------------
                       condition |
No Health Warning Label Control  |   .8999767   .0217685    41.34   0.000     .8573112    .9426422
             DrinkWise Control   |   .8960127   .0228582    39.20   0.000     .8512115    .9408139
                     Text-Only   |   .8835057   .0231529    38.16   0.000     .8381268    .9288846
              Text + Pictogram   |   .9145721   .0185436    49.32   0.000     .8782274    .9509168
             Text + Photograph   |   .9234341   .0179647    51.40   0.000     .8882238    .9586443
-------------------------------------------------------------------------------------------------

.
.         ** Interaction
.
.         *** Simple model
.         logistic FD2_FD6_2_b i.condition##i.RET_sum_b ///
>                 $covariates     ///
>                 if FUP_compl == 1,      ///
>                 vce(robust)

Logistic regression                              Number of obs     =      1,086
                                                 Wald chi2(20)     =      42.78
                                                 Prob > chi2       =     0.0022
Log pseudolikelihood = -369.28365                Pseudo R2         =     0.0476

------------------------------------------------------------------------------------------------------------
                                          |               Robust
                              FD2_FD6_2_b | Odds Ratio   Std. Err.      z    P>|z|     [95% Conf. Interval]
------------------------------------------+-----------------------------------------------------------------
                                condition |
                       DrinkWise Control  |   .8420576   .3760314    -0.38   0.700     .3509354    2.020489
                               Text-Only  |   .7014921   .3023213    -0.82   0.411     .3014264    1.632541
                        Text + Pictogram  |   1.551273   .8011553     0.85   0.395     .5637478    4.268659
                       Text + Photograph  |   1.359576   .6732304     0.62   0.535     .5151198    3.588382
                                          |
                                RET_sum_b |
                    High (6-8 exposures)  |    1.28507   .5589658     0.58   0.564     .5478738    3.014208
                                          |
                      condition#RET_sum_b |
    DrinkWise Control#High (6-8 exposures)  |    1.26178   .7688032     0.38   0.703     .3822486    4.165059
            Text-Only#High (6-8 exposures)  |   1.412809   .8346216     0.58   0.559     .4438473     4.49711
     Text + Pictogram#High (6-8 exposures)  |   .6520919   .4244298    -0.66   0.511      .182089    2.335253
    Text + Photograph#High (6-8 exposures)  |   .9591086   .6121321    -0.07   0.948     .2745398     3.35066
                                          |
                          Quota_Age_Group |
                                   30-49  |   1.177784   .3026888     0.64   0.524     .7117185    1.949051
                                   50-69  |   .9761414   .2785034    -0.08   0.933      .558028    1.707534
                                          |
                               gender_A2b |
                                Not male  |   1.571901   .3783601     1.88   0.060     .9807063    2.519482
                                          |
                                 educ_G2b |
                      Tertiary education  |   .8062774   .2118815    -0.82   0.413     .4817231    1.349496
                                          |
                               parent_G3r |
                                     Yes  |   1.249448   .2776311     1.00   0.316     .8083114    1.931336
                                          |
                             seifa_quin_b |
                                 41-100%  |   1.070621   .2280194     0.32   0.749      .705258    1.625263
                                          |
                              NHMRCrisk_b |
                High either STH LTH or Both  |   .8833758   .2094306    -0.52   0.601     .5550636     1.40588
                                          |
                                    B17_r |
Definitely/Probably drink more than should  |   1.402197   .3222423     1.47   0.141     .8937009    2.200015
                                          |
                                     C1_b |
                                    Wine  |    2.14728    .591636     2.77   0.006     1.251292     3.68484
                                 Spirits  |   1.570199   .3930364     1.80   0.071     .9613701    2.564596
                                          |
                                base_7day |   1.009276   .0082571     1.13   0.259     .9932214     1.02559
                                    _cons |   2.940805   1.483677     2.14   0.033     1.094015    7.905135
------------------------------------------------------------------------------------------------------------
Note: _cons estimates baseline odds.

.
.         **** Omnibus test for interaction
.         testparm i.condition#i.RET_sum_b

 ( 1)  [FD2_FD6_2_b]2.condition#1.RET_sum_b = 0
 ( 2)  [FD2_FD6_2_b]3.condition#1.RET_sum_b = 0
 ( 3)  [FD2_FD6_2_b]4.condition#1.RET_sum_b = 0
 ( 4)  [FD2_FD6_2_b]5.condition#1.RET_sum_b = 0

           chi2(  4) =    1.70
         Prob > chi2 =    0.7904
```

```
. di"Increase your risk of heart disease"
Increase your risk of heart disease

.
.
.         ** Logistic regression
.
.         *** Simple model
.         logistic FD2_FD6_4_b i.condition ///
>                $covariates     ///
>                if FUP_compl == 1,      ///
>                vce(robust)

Logistic regression                             Number of obs     =      1,086
                                                Wald chi2(15)     =      38.42
                                                Prob > chi2       =     0.0008
Log pseudolikelihood = -553.81425               Pseudo R2         =     0.0323

--------------------------------------------------------------------------------------------------------------
                                             |               Robust
                                 FD2_FD6_4_b | Odds Ratio   Std. Err.      z    P>|z|     [95% Conf. Interval]
---------------------------------------------+----------------------------------------------------------------
                                   condition |
                           DrinkWise Control |   .7059109   .1657101    -1.48   0.138     .4455878    1.118321
                                   Text-Only |    .919467   .2188553    -0.35   0.724     .5766731    1.466029
                            Text + Pictogram |   1.116277   .2793089     0.44   0.660     .6835797    1.822868
                           Text + Photograph |   .9351817   .2206663    -0.28   0.776     .5889056    1.485068
                                             |
                             Quota_Age_Group |
                                       30-49 |   1.071789   .2058319     0.36   0.718     .7355974    1.561631
                                       50-69 |   .7263729   .1468318    -1.58   0.114     .4887587    1.079505
                                             |
                                  gender_A2b |
                                    Not male |    1.44233   .2460566     2.15   0.032     1.032411    2.015008
                                             |
                                    educ_G2b |
                          Tertiary education |   .7980464    .155806    -1.16   0.248     .5443076     1.17007
                                             |
                                  parent_G3r |
                                         Yes |   1.254503   .2053504     1.39   0.166     .9102024    1.729041
                                             |
                                seifa_quin_b |
                                     41-100% |   .9311869   .1565958    -0.42   0.672      .669718    1.294737
                                             |
                                 NHMRCrisk_b |
                  High either STH LTH or Both |   .8163802   .1483322    -1.12   0.264     .5717858    1.165605
                                             |
                                       B17_r |
     Definitely/Probably drink more than should |   1.585829   .2823238     2.59   0.010      1.11871    2.247993
                                             |
                                        C1_b |
                                        Wine |   1.446323   .2857946     1.87   0.062     .9818995    2.130411
                                     Spirits |   1.498459   .2921101     2.07   0.038     1.022613    2.195727
                                             |
                                   base_7day |   1.001526   .0055931     0.27   0.785     .9906232    1.012548
                                       _cons |   2.818105   .9822968     2.97   0.003      1.42317    5.580299
--------------------------------------------------------------------------------------------------------------
Note: _cons estimates baseline odds.

.
.         **** Omnibus test
.         testparm i.condition

 ( 1)  [FD2_FD6_4_b]2.condition = 0
 ( 2)  [FD2_FD6_4_b]3.condition = 0
 ( 3)  [FD2_FD6_4_b]4.condition = 0
 ( 4)  [FD2_FD6_4_b]5.condition = 0

           chi2(  4) =    4.13
         Prob > chi2 =    0.3881

.
.         **** Adjusted means/proportions
.         margins i.condition, asbal atmeans

Adjusted predictions                            Number of obs     =      1,086
Model VCE    : Robust

Expression   : Pr(FD2_FD6_4_b), predict()
at           : condition                    (asbalanced)
               Quota_Age_Group              (asbalanced)
               gender_A2b                   (asbalanced)
               educ_G2b                     (asbalanced)
               parent_G3r                   (asbalanced)
               seifa_quin_b                 (asbalanced)
               NHMRCrisk_b                  (asbalanced)
               B17_r                        (asbalanced)
               C1_b                         (asbalanced)
               base_7day       =    14.63444 (mean)

---------------------------------------------------------------------------------------------------
                                  |            Delta-method
                                  |     Margin   Std. Err.      z    P>|z|     [95% Conf. Interval]
----------------------------------+----------------------------------------------------------------
                        condition |
No Health Warning Label Control |   .8190485   .0278404    29.42   0.000     .7644823    .8736147
                DrinkWise Control |   .7616321   .0323226    23.56   0.000      .698281    .8249832
                        Text-Only |     .80627   .0280301    28.76   0.000      .751332     .861208
                 Text + Pictogram |   .8347832   .0261804    31.89   0.000     .7834707    .8860958
                Text + Photograph |   .8089033    .027741    29.16   0.000      .754532    .8632746
---------------------------------------------------------------------------------------------------

.
```

```
.         ** Interaction
.
.         *** Linear regression
.         logistic FD2_FD6_4_b i.condition##i.RET_sum_b ///
>                 $covariates     ///
>                 if FUP_compl == 1,      ///
>                 vce(robust)
```

```
Logistic regression                               Number of obs     =      1,086
                                                  Wald chi2(20)     =      43.65
                                                  Prob > chi2       =     0.0017
Log pseudolikelihood =  -550.4277                 Pseudo R2         =     0.0383
```

FD2_FD6_4_b	Odds Ratio	Robust Std. Err.	z	P>|z|	[95% Conf.	Interval]
condition						
DrinkWise Control	.6040615	.23077	-1.32	0.187	.2856907	1.277221
Text-Only	.6722605	.2545501	-1.05	0.294	.3200603	1.412028
Text + Pictogram	.8092699	.3192707	-0.54	0.592	.3734909	1.753504
Text + Photograph	.8579196	.3446774	-0.38	0.703	.3903626	1.885493
RET_sum_b						
High (6-8 exposures)	1.064087	.3899538	0.17	0.865	.5188469	2.182304
condition#RET_sum_b						
DrinkWise Control#High (6-8 exposures)	1.285763	.6249871	0.52	0.605	.4959144	3.333613
Text-Only#High (6-8 exposures)	1.726537	.8509658	1.11	0.268	.6571135	4.536399
Text + Pictogram#High (6-8 exposures)	1.704688	.863333	1.05	0.292	.631767	4.599735
Text + Photograph#High (6-8 exposures)	1.131203	.5638718	0.25	0.805	.425835	3.004967
Quota_Age_Group						
30-49	.9768428	.1924391	-0.12	0.905	.6639526	1.437184
50-69	.6604847	.1374628	-1.99	0.046	.4392453	.9931581
gender_A2b						
Not male	1.442531	.2461231	2.15	0.032	1.03251	2.015377
educ_G2b						
Tertiary education	.7985258	.1562123	-1.15	0.250	.5442167	1.171672
parent_G3r						
Yes	1.237518	.2053151	1.28	0.199	.8939837	1.713063
seifa_quin_b						
41-100%	.9110959	.1548494	-0.55	0.584	.6529716	1.271259
NHMRCrisk_b						
High either STH LTH or Both	.7917466	.1451128	-1.27	0.203	.5528106	1.133956
B17_r						
Definitely/Probably drink more than should	1.612532	.2873975	2.68	0.007	1.137105	2.286735
C1_b						
Wine	1.425576	.2822918	1.79	0.073	.9670209	2.101575
Spirits	1.523241	.2953741	2.17	0.030	1.041624	2.227545
base_7day	1.001536	.0056659	0.27	0.786	.9904924	1.012703
_cons	2.955633	1.1775	2.72	0.007	1.35375	6.45301

Note: _cons estimates baseline odds.

```
.
.         **** Omnibus test for interaction
.         testparm i.condition#i.RET_sum_b

 ( 1)  [FD2_FD6_4_b]2.condition#1.RET_sum_b = 0
 ( 2)  [FD2_FD6_4_b]3.condition#1.RET_sum_b = 0
 ( 3)  [FD2_FD6_4_b]4.condition#1.RET_sum_b = 0
 ( 4)  [FD2_FD6_4_b]5.condition#1.RET_sum_b = 0

           chi2(  4) =    1.94
         Prob > chi2 =    0.7462
```

```
. di "Increase your risk of pregnancy complications"
Increase your risk of pregnancy complications

.
.         ** Logistic regression
.
.         *** Simple model
.         logistic FD2_FD6_5_b i.condition ///
>                 $covariates     ///
>                 if FUP_compl == 1,      ///
>                 vce(robust)

Logistic regression                             Number of obs     =      1,086
                                                Wald chi2(15)     =      38.76
                                                Prob > chi2       =     0.0007
Log pseudolikelihood = -492.53225               Pseudo R2         =     0.0365

--------------------------------------------------------------------------------------------------------------
                                             |               Robust
                                 FD2_FD6_5_b | Odds Ratio   Std. Err.      z    P>|z|     [95% Conf. Interval]
---------------------------------------------+----------------------------------------------------------------
                                   condition |
                            DrinkWise Control |    1.02301   .2733575     0.09   0.932     .6059419    1.727144
                                    Text-Only |   .9263715   .2438223    -0.29   0.771     .5530288    1.551753
                             Text + Pictogram |   .9053548   .2462792    -0.37   0.715     .5312154    1.543004
                            Text + Photograph |   .6536561   .1664465    -1.67   0.095     .3968259     1.07671
                                             |
                             Quota_Age_Group |
                                        30-49 |   .9517528   .1941915    -0.24   0.809     .6380412     1.41971
                                        50-69 |   1.095344   .2642336     0.38   0.706     .6826726    1.757474
                                             |
                                  gender_A2b |
                                     Not male |   1.313209   .2516913     1.42   0.155     .9019686    1.911949
                                             |
                                    educ_G2b |
                           Tertiary education |   .9278556   .1933784    -0.36   0.719     .6167054    1.395992
                                             |
                                  parent_G3r |
                                          Yes |   1.141245   .2077725     0.73   0.468     .7987501    1.630597
                                             |
                                seifa_quin_b |
                                      41-100% |   1.346494   .2343207     1.71   0.087      .957361    1.893794
                                             |
                                 NHMRCrisk_b |
                     High either STH LTH or Both |   .9218306   .1829748    -0.41   0.682     .6247346    1.360212
                                             |
                                       B17_r |
Definitely/Probably drink more than should |   1.098562   .1996957     0.52   0.605      .769297    1.568756
                                             |
                                        C1_b |
                                         Wine |   2.185066   .4904598     3.48   0.000     1.407356    3.392542
                                      Spirits |   1.594066   .3240538     2.29   0.022     1.070207    2.374352
                                             |
                                   base_7day |   .9973014   .0057328    -0.47   0.638     .9861282    1.008601
                                       _cons |   2.673601   .9743385     2.70   0.007     1.308862    5.461342
--------------------------------------------------------------------------------------------------------------
Note: _cons estimates baseline odds.

.
.         **** Omnibus test
.         testparm i.condition

 ( 1)  [FD2_FD6_5_b]2.condition = 0
 ( 2)  [FD2_FD6_5_b]3.condition = 0
 ( 3)  [FD2_FD6_5_b]4.condition = 0
 ( 4)  [FD2_FD6_5_b]5.condition = 0

           chi2(  4) =    4.54
         Prob > chi2 =    0.3380

.
.         **** Adjusted means/proportions
.         margins i.condition, asbal atmeans

Adjusted predictions                            Number of obs     =      1,086
Model VCE    : Robust

Expression   : Pr(FD2_FD6_5_b), predict()
at           : condition                   (asbalanced)
               Quota_Age_Group             (asbalanced)
               gender_A2b                  (asbalanced)
               educ_G2b                    (asbalanced)
               parent_G3r                  (asbalanced)
               seifa_quin_b                (asbalanced)
               NHMRCrisk_b                 (asbalanced)
               B17_r                       (asbalanced)
               C1_b                        (asbalanced)
               base_7day       =    14.63444 (mean)

-----------------------------------------------------------------------------------------------
                                  |            Delta-method
                                  |     Margin   Std. Err.      z    P>|z|     [95% Conf. Interval]
----------------------------------+------------------------------------------------------------
                        condition |
No Health Warning Label Control |   .8447028   .0279092    30.27   0.000     .7900018    .8994038
                DrinkWise Control |   .8476637   .0247858    34.20   0.000     .7990844    .8962429
                        Text-Only |   .8344037   .0269356    30.98   0.000     .7816108    .8871965
                 Text + Pictogram |   .8312084   .0287368    28.92   0.000     .7748853    .8875315
                Text + Photograph |   .7804808   .0310315    25.15   0.000     .7196602    .8413013
-----------------------------------------------------------------------------------------------
```

```
.         ** Interaction
.
.         *** Simple model
.         logistic FD2_FD6_5_b i.condition##i.RET_sum_b ///
>                 $covariates ///
>                 if FUP_compl == 1,      ///
>                 vce(robust)

Logistic regression                                 Number of obs     =      1,086
                                                    Wald chi2(20)     =      40.90
                                                    Prob > chi2       =     0.0038
Log pseudolikelihood = -490.84745                   Pseudo R2         =     0.0398

-----------------------------------------------------------------------------------------------------------------
                                                |               Robust
                                    FD2_FD6_5_b | Odds Ratio   Std. Err.      z    P>|z|     [95% Conf. Interval]
------------------------------------------------+----------------------------------------------------------------
                                      condition |
                             DrinkWise Control  |   1.046964   .4312878     0.11   0.911     .4669674    2.347345
                                     Text-Only  |   .8398698   .3359977    -0.44   0.663     .3834254    1.839683
                              Text + Pictogram  |   1.003355    .427909     0.01   0.994     .4349462    2.314587
                             Text + Photograph  |   .7136772   .2959746    -0.81   0.416     .3165873     1.60883
                                                |
                                      RET_sum_b |
                          High (6-8 exposures)  |   1.383597   .5629354     0.80   0.425     .6232846    3.071373
                                                |
                            condition#RET_sum_b |
        DrinkWise Control#High (6-8 exposures)  |   .9635863   .5274687    -0.07   0.946     .3295654    2.817343
                Text-Only#High (6-8 exposures)  |   1.203128   .6490629     0.34   0.732     .4179327     3.46352
         Text + Pictogram#High (6-8 exposures)  |   .8409097   .4629443    -0.31   0.753     .2858506    2.473772
        Text + Photograph#High (6-8 exposures)  |   .8485695   .4466238    -0.31   0.755     .3024672    2.380656
                                                |
                                Quota_Age_Group |
                                        30-49   |   .8835632   .1885259    -0.58   0.562     .5815897    1.342328
                                        50-69   |   1.017159    .252389     0.07   0.945     .6254302     1.65424
                                                |
                                     gender_A2b |
                                     Not male   |   1.319195   .2529819     1.44   0.149     .9058876    1.921073
                                                |
                                       educ_G2b |
                           Tertiary education   |   .9331925    .195585    -0.33   0.741     .6188288    1.407252
                                                |
                                     parent_G3r |
                                          Yes   |    1.12675   .2063957     0.65   0.515     .7868754    1.613425
                                                |
                                   seifa_quin_b |
                                      41-100%   |   1.332955   .2340255     1.64   0.102     .9448671    1.880442
                                                |
                                    NHMRCrisk_b |
                  High either STH LTH or Both   |   .9066555   .1800349    -0.49   0.622     .6143543    1.338029
                                                |
                                          B17_r |
      Definitely/Probably drink more than should|   1.105177   .2026353     0.55   0.585     .7715484    1.583071
                                                |
                                           C1_b |
                                         Wine   |    2.15681   .4850419     3.42   0.001      1.38799    3.351486
                                      Spirits   |     1.5991   .3261277     2.30   0.021     1.072205    2.384919
                                                |
                                      base_7day |    .997521   .0056339    -0.44   0.660     .9865397    1.008625
                                          _cons |    2.32132   .9888524     1.98   0.048     1.007243    5.349779
-----------------------------------------------------------------------------------------------------------------
Note: _cons estimates baseline odds.

.
.         **** Omnibus test for interaction
.         testparm i.condition#i.RET_sum_b

 ( 1)  [FD2_FD6_5_b]2.condition#1.RET_sum_b = 0
 ( 2)  [FD2_FD6_5_b]3.condition#1.RET_sum_b = 0
 ( 3)  [FD2_FD6_5_b]4.condition#1.RET_sum_b = 0
 ( 4)  [FD2_FD6_5_b]5.condition#1.RET_sum_b = 0

           chi2(  4) =    0.64
         Prob > chi2 =    0.9589
```

```
. di "Negative emotional arousal"
Negative emotional arousal

.
.         ** Logistic regression
.
.         *** Linear regression
.         regress neg_ar i.condition ///
>                 $covariates     ///
>                 if FUP_compl == 1,      ///
>                 vce(hc3)

Linear regression                               Number of obs     =      1,086
                                                F(15, 1070)       =      22.29
                                                Prob > F          =     0.0000
                                                R-squared         =     0.1982
                                                Root MSE          =     1.3849

--------------------------------------------------------------------------------------------------------------
                                             |             Robust HC3
                                      neg_ar |      Coef.   Std. Err.      t    P>|t|     [95% Conf. Interval]
---------------------------------------------+----------------------------------------------------------------
                                   condition |
                           DrinkWise Control |  -.0172355   .1183804    -0.15   0.884    -.2495195    .2150484
                                   Text-Only |   .9278281   .1352043     6.86   0.000     .6625325    1.193124
                            Text + Pictogram |   1.167013   .1359341     8.59   0.000     .9002848     1.43374
                           Text + Photograph |   1.332357   .1366632     9.75   0.000     1.064199    1.600516
                                             |
                             Quota_Age_Group |
                                       30-49 |  -.3970216   .1188105    -3.34   0.001    -.6301497   -.1638935
                                       50-69 |  -.8279297   .1214112    -6.82   0.000    -1.066161   -.5896987
                                             |
                                  gender_A2b |
                                    Not male |  -.1418477   .1016601    -1.40   0.163    -.3413234    .0576281
                                             |
                                   educ_G2b |
                         Tertiary education |  -.0579472   .1057635    -0.55   0.584    -.2654746    .1495802
                                             |
                                  parent_G3r |
                                         Yes |   .1533314   .0986591     1.55   0.120    -.0402558    .3469186
                                             |
                                seifa_quin_b |
                                     41-100% |   .1323073   .0915119     1.45   0.149    -.0472559    .3118705
                                             |
                                 NHMRCrisk_b |
                  High either STH LTH or Both |  -.2087698   .1027051    -2.03   0.042    -.4102962   -.0072435
                                             |
                                       B17_r |
   Definitely/Probably drink more than should |   .1489471   .0964864     1.54   0.123     -.040377    .3382711
                                             |
                                        C1_b |
                                        Wine |   .0684551   .1171794     0.58   0.559    -.1614724    .2983826
                                     Spirits |  -.4563884   .1095617    -4.17   0.000    -.6713685   -.2414083
                                             |
                                  base_7day |  -.0010737   .0030774    -0.35   0.727    -.0071121    .0049648
                                       _cons |   2.310982   .1913675    12.08   0.000     1.935484     2.68648
--------------------------------------------------------------------------------------------------------------

.
.         **** Omnibus test
.         testparm i.condition

 ( 1)  2.condition = 0
 ( 2)  3.condition = 0
 ( 3)  4.condition = 0
 ( 4)  5.condition = 0

       F(  4,  1070) =   49.96
            Prob > F =    0.0000

.
.         **** Adjusted means/proportions
.         margins i.condition, asbal atmeans

Adjusted predictions                            Number of obs     =      1,086
Model VCE    : Robust HC3

Expression   : Linear prediction, predict()
at           : condition                    (asbalanced)
               Quota_Age_Group              (asbalanced)
               gender_A2b                   (asbalanced)
               educ_G2b                     (asbalanced)
               parent_G3r                   (asbalanced)
               seifa_quin_b                 (asbalanced)
               NHMRCrisk_b                  (asbalanced)
               B17_r                        (asbalanced)
               C1_b                         (asbalanced)
               base_7day       =    14.63444 (mean)

---------------------------------------------------------------------------------------------------
                                  |            Delta-method
                                  |     Margin   Std. Err.      t    P>|t|     [95% Conf. Interval]
----------------------------------+----------------------------------------------------------------
                        condition |
  No Health Warning Label Control |   1.770652   .0978949    18.09   0.000     1.578564     1.96274
                DrinkWise Control |   1.753416   .0830867    21.10   0.000     1.590385    1.916448
                        Text-Only |    2.69848   .1076945    25.06   0.000     2.487164    2.909796
                 Text + Pictogram |   2.937665   .1061533    27.67   0.000     2.729372    3.145957
                Text + Photograph |   3.103009   .1109913    27.96   0.000     2.885224    3.320795
---------------------------------------------------------------------------------------------------
```

```
.         ** Interaction
.
.         *** Linear regression
.         regress neg_ar i.condition##i.RET_sum_b ///
>                 $covariates     ///
>                 if FUP_compl == 1,      ///
>                 vce(hc3)
```

```
Linear regression                               Number of obs     =      1,086
                                                F(20, 1065)       =      18.91
                                                Prob > F          =     0.0000
                                                R-squared         =     0.2091
                                                Root MSE          =     1.3787
```

neg_ar	Coef.	Robust HC3 Std. Err.	t	P>|t|	[95% Conf.	Interval]
**condition**						
DrinkWise Control	-.0106624	.2267684	-0.05	0.963	-.455626	.4343011
Text-Only	.5772558	.2460482	2.35	0.019	.0944615	1.06005
Text + Pictogram	.8132995	.253801	3.20	0.001	.3152928	1.311306
Text + Photograph	.8234354	.2519444	3.27	0.001	.3290716	1.317799
**RET_sum_b**						
High (6-8 exposures)	-.4736515	.201101	-2.36	0.019	-.8682507	-.0790524
**condition#RET_sum_b**						
DrinkWise Control#High (6-8 exposures)	.0011617	.2626874	0.00	0.996	-.514282	.5166053
Text-Only#High (6-8 exposures)	.5656017	.2910462	1.94	0.052	-.0054873	1.136691
Text + Pictogram#High (6-8 exposures)	.565725	.2985532	1.89	0.058	-.0200942	1.151544
Text + Photograph#High (6-8 exposures)	.7925111	.2975524	2.66	0.008	.2086555	1.376367
**Quota_Age_Group**						
30-49	-.3753123	.120055	-3.13	0.002	-.6108835	-.1397412
50-69	-.8060134	.1217867	-6.62	0.000	-1.044983	-.5670443
**gender_A2b**						
Not male	-.1428926	.1012051	-1.41	0.158	-.3414767	.0556915
**educ_G2b**						
Tertiary education	-.0554779	.1052572	-0.53	0.598	-.262013	.1510572
**parent_G3r**						
Yes	.1477909	.0990254	1.49	0.136	-.0465162	.342098
**seifa_quin_b**						
41-100%	.1235781	.0912511	1.35	0.176	-.0554742	.3026304
**NHMRCrisk_b**						
High either STH LTH or Both	-.2084985	.1020555	-2.04	0.041	-.4087512	-.0082459
**B17_r**						
Definitely/Probably drink more than should	.1458654	.0956687	1.52	0.128	-.0418552	.333586
**C1_b**						
Wine	.0836195	.1166802	0.72	0.474	-.1453296	.3125686
Spirits	-.4492333	.1093574	-4.11	0.000	-.6638137	-.234653
base_7day	-.0012111	.0029948	-0.40	0.686	-.0070875	.0046653
_cons	2.596674	.2466687	10.53	0.000	2.112663	3.080686

```
.
.         **** Omnibus test for interaction
.         testparm i.condition#i.RET_sum_b

 ( 1)  2.condition#1.RET_sum_b = 0
 ( 2)  3.condition#1.RET_sum_b = 0
 ( 3)  4.condition#1.RET_sum_b = 0
 ( 4)  5.condition#1.RET_sum_b = 0

       F(  4,  1065) =    3.35
            Prob > F =    0.0099

.
```

```
. di "Positive emotional arousal"
Positive emotional arousal

.
.         *** Linear regression
.         regress pos_ar i.condition ///
>                 $covariates     ///
>                 if FUP_compl == 1,      ///
>                 vce(hc3)

Linear regression                               Number of obs     =      1,086
                                                F(15, 1070)       =       9.68
                                                Prob > F          =     0.0000
                                                R-squared         =     0.1124
                                                Root MSE          =     1.4743
```

pos_ar	Coef.	Robust HC3 Std. Err.	t	P>|t|	[95% Conf.	Interval]
condition						
DrinkWise Control	-.2131562	.1463552	-1.46	0.146	-.500332	.0740197
Text-Only	-.5465061	.144575	-3.78	0.000	-.8301888	-.2628233
Text + Pictogram	-.4019307	.1410994	-2.85	0.004	-.6787936	-.1250679
Text + Photograph	-.7208618	.1494939	-4.82	0.000	-1.014196	-.4275274
Quota_Age_Group						
30-49	-.4426639	.1255961	-3.52	0.000	-.6891065	-.1962214
50-69	-.9990384	.1284695	-7.78	0.000	-1.251119	-.7469576
gender_A2b						
Not male	-.2081605	.1083726	-1.92	0.055	-.4208074	.0044865
educ_G2b						
Tertiary education	-.1588226	.1137662	-1.40	0.163	-.3820527	.0644075
parent_G3r						
Yes	.0844032	.1040727	0.81	0.418	-.1198066	.288613
seifa_quin_b						
41-100%	-.1001702	.1016711	-0.99	0.325	-.2996675	.0993271
NHMRCrisk_b						
High either STH LTH or Both	-.0045559	.1077264	-0.04	0.966	-.2159348	.206823
B17_r						
Definitely/Probably drink more than should	-.0707086	.1055128	-0.67	0.503	-.277744	.1363268
C1_b						
Wine	-.458306	.1217381	-3.76	0.000	-.6971784	-.2194335
Spirits	-.4772578	.1231698	-3.87	0.000	-.7189395	-.235576
base_7day	.0019298	.0037422	0.52	0.606	-.005413	.0092727
_cons	4.258536	.2005323	21.24	0.000	3.865054	4.652017

```
.
.         **** Omnibus test
.         testparm i.condition

 ( 1)  2.condition = 0
 ( 2)  3.condition = 0
 ( 3)  4.condition = 0
 ( 4)  5.condition = 0

       F(  4,  1070) =    7.29
            Prob > F =    0.0000

.
.         **** Adjusted means/proportions
.         margins i.condition, asbal atmeans

Adjusted predictions                            Number of obs     =      1,086
Model VCE    : Robust HC3

Expression   : Linear prediction, predict()
at           : condition                     (asbalanced)
               Quota_Age_Group               (asbalanced)
               gender_A2b                    (asbalanced)
               educ_G2b                      (asbalanced)
               parent_G3r                    (asbalanced)
               seifa_quin_b                  (asbalanced)
               NHMRCrisk_b                   (asbalanced)
               B17_r                         (asbalanced)
               C1_b                          (asbalanced)
               base_7day       =    14.63444 (mean)
```

	Margin	Delta-method Std. Err.	t	P>|t|	[95% Conf.	Interval]
condition						
No Health Warning Label Control	3.265348	.1155963	28.25	0.000	3.038527	3.492169
DrinkWise Control	3.052192	.1085634	28.11	0.000	2.839171	3.265213
Text-Only	2.718842	.1059529	25.66	0.000	2.510943	2.926741
Text + Pictogram	2.863417	.0992008	28.86	0.000	2.668767	3.058068
Text + Photograph	2.544486	.1137303	22.37	0.000	2.321327	2.767646

```
.
.         ** Interaction
.
.         *** Linear regression
.         regress pos_ar i.condition##i.RET_sum_b ///
>                 $covariates     ///
>                 if FUP_compl == 1,      ///
>                 vce(hc3)

Linear regression                                Number of obs     =      1,086
                                                 F(20, 1065)       =       7.27
                                                 Prob > F          =     0.0000
                                                 R-squared         =     0.1135
                                                 Root MSE          =     1.4769

--------------------------------------------------------------------------------------------------------------
                                             |             Robust HC3
                                      pos_ar |      Coef.   Std. Err.      t    P>|t|     [95% Conf. Interval]
---------------------------------------------+----------------------------------------------------------------
                                   condition |
                          DrinkWise Control  |  -.1896427    .244702    -0.77   0.439    -.6697956    .2905101
                                   Text-Only |  -.4195397    .241826    -1.73   0.083    -.8940492    .0549699
                            Text + Pictogram |  -.3659442    .235194    -1.56   0.120    -.8274405    .0955521
                           Text + Photograph |  -.5369453   .2600606    -2.06   0.039    -1.047235   -.0266559
                                             |
                                   RET_sum_b |
                        High (6-8 exposures) |   .1193871   .2236837     0.53   0.594    -.3195237    .5582979
                                             |
                         condition#RET_sum_b |
    DrinkWise Control#High (6-8 exposures)   |  -.0402647   .3060681    -0.13   0.895    -.6408297    .5603003
            Text-Only#High (6-8 exposures)   |  -.2061948   .3018019    -0.68   0.495    -.7983888    .3859991
     Text + Pictogram#High (6-8 exposures)   |  -.0591234    .294619    -0.20   0.841    -.6372231    .5189762
    Text + Photograph#High (6-8 exposures)   |  -.2822605   .3179981    -0.89   0.375    -.9062345    .3417135
                                             |
                             Quota_Age_Group |
                                      30-49  |  -.4451584   .1286078    -3.46   0.001    -.6975119   -.1928049
                                      50-69  |  -1.002558   .1309675    -7.66   0.000    -1.259541   -.7455741
                                             |
                                  gender_A2b |
                                   Not male  |  -.2090519   .1089616    -1.92   0.055    -.4228557    .0047519
                                             |
                                    educ_G2b |
                         Tertiary education  |  -.1614296   .1144648    -1.41   0.159    -.3860318    .0631727
                                             |
                                  parent_G3r |
                                        Yes  |   .0874306    .105319     0.83   0.407    -.1192256    .2940868
                                             |
                                 seifa_quin_b |
                                    41-100%  |  -.0947182    .102738    -0.92   0.357      -.29631    .1068737
                                             |
                                  NHMRCrisk_b |
                   High either STH LTH or Both |  -.0040977   .1091875    -0.04   0.970    -.2183448    .2101495
                                             |
                                       B17_r |
Definitely/Probably drink more than should   |  -.0671579   .1060093    -0.63   0.527    -.2751686    .1408529
                                             |
                                        C1_b |
                                       Wine  |  -.4612539    .122187    -3.77   0.000    -.7010086   -.2214993
                                    Spirits  |  -.4792005   .1234125    -3.88   0.000    -.7213598   -.2370413
                                             |
                                   base_7day |   .0019205   .0037839     0.51   0.612    -.0055042    .0093453
                                       _cons |   4.183119   .2455305    17.04   0.000      3.70134    4.664897
--------------------------------------------------------------------------------------------------------------

.
.         **** Omnibus test for interaction
.         testparm i.condition#i.RET_sum_b

 ( 1)  2.condition#1.RET_sum_b = 0
 ( 2)  3.condition#1.RET_sum_b = 0
 ( 3)  4.condition#1.RET_sum_b = 0
 ( 4)  5.condition#1.RET_sum_b = 0

       F(  4,  1065) =    0.30
            Prob > F =    0.8768
```

```
. di "Show/talk images + others"
Show/talk images + others

.
.         ** Logistic regression
.
.         *** Simple model
.         logistic show_talk i.condition ///
>                 $covariates  ///
>                 if FUP_compl == 1,      ///
>                 vce(robust)

Logistic regression                             Number of obs     =      1,086
                                                Wald chi2(15)     =      33.92
                                                Prob > chi2       =     0.0035
Log pseudolikelihood = -507.00304               Pseudo R2         =     0.0336
```

show_talk	Odds Ratio	Robust Std. Err.	z	P>|z|	[95% Conf.	Interval]
condition						
DrinkWise Control	.6736738	.1801452	-1.48	0.140	.3988708	1.137803
Text-Only	1.139272	.2788774	0.53	0.594	.7051243	1.840727
Text + Pictogram	1.172385	.2946951	0.63	0.527	.716323	1.918807
Text + Photograph	.9880177	.251177	-0.05	0.962	.6003016	1.626147
Quota_Age_Group						
30-49	.6016533	.1293084	-2.36	0.018	.3948249	.9168284
50-69	.4547732	.1089492	-3.29	0.001	.2843637	.7273034
gender_A2b						
Not male	.8171071	.1507426	-1.09	0.274	.5691759	1.173036
educ_G2b						
Tertiary education	.7049201	.1350138	-1.83	0.068	.4842936	1.026056
parent_G3r						
Yes	1.27077	.2326047	1.31	0.190	.8876893	1.819168
seifa_quin_b						
41-100%	.8927608	.1536787	-0.66	0.510	.637104	1.251007
NHMRCrisk_b						
High either STH LTH or Both	1.495892	.2918626	2.06	0.039	1.020523	2.192692
B17_r						
Definitely/Probably drink more than should	1.094314	.1927812	0.51	0.609	.7747989	1.545592
C1_b						
Wine	.9478942	.1934022	-0.26	0.793	.635457	1.413948
Spirits	.6181853	.1341697	-2.22	0.027	.4039946	.945936
base_7day	.9927794	.0056754	-1.27	0.205	.9817179	1.003966
_cons	.4292613	.146119	-2.48	0.013	.2202804	.8365033

```
Note: _cons estimates baseline odds.

.
.         **** Omnibus test
.         testparm i.condition

 ( 1)  [show_talk]2.condition = 0
 ( 2)  [show_talk]3.condition = 0
 ( 3)  [show_talk]4.condition = 0
 ( 4)  [show_talk]5.condition = 0

           chi2(  4) =    5.53
         Prob > chi2 =    0.2370
```

```
.         **** Adjusted means/proportions
.         margins i.condition, asbal atmeans

Adjusted predictions                             Number of obs     =      1,086
Model VCE    : Robust

Expression   : Pr(show_talk), predict()
at           : condition                   (asbalanced)
               Quota_Age_Group             (asbalanced)
               gender_A2b                  (asbalanced)
               educ_G2b                    (asbalanced)
               parent_G3r                  (asbalanced)
               seifa_quin_b                (asbalanced)
               NHMRCrisk_b                 (asbalanced)
               B17_r                       (asbalanced)
               C1_b                        (asbalanced)
               base_7day       =    14.63444 (mean)

----------------------------------------------------------------------------------------------
                                  |            Delta-method
                                  |     Margin   Std. Err.      z    P>|z|     [95% Conf. Interval]
----------------------------------+-----------------------------------------------------------
                        condition |
No Health Warning Label Control   |   .1782541   .0292287     6.10   0.000     .1209668    .2355414
                DrinkWise Control |   .1275018    .022077     5.78   0.000     .0842315     .170772
                        Text-Only |   .1981604   .0297407     6.66   0.000     .1398697    .2564512
                 Text + Pictogram |   .2027522    .030345     6.68   0.000     .1432771    .2622272
               Text + Photograph  |   .1764952   .0279811     6.31   0.000     .1216531    .2313372
----------------------------------------------------------------------------------------------

.
.         ** Interaction
.
.         *** Simple model
.         logistic show_talk i.condition##i.RET_sum_b ///
>                 $covariates     ///
>                 if FUP_compl == 1,      ///
>                 vce(robust)

Logistic regression                              Number of obs     =      1,086
                                                 Wald chi2(20)     =      44.67
                                                 Prob > chi2       =     0.0012
Log pseudolikelihood = -502.82253                Pseudo R2         =     0.0416

-----------------------------------------------------------------------------------------------------------
                                          |               Robust
                                show_talk | Odds Ratio   Std. Err.      z    P>|z|     [95% Conf. Interval]
------------------------------------------+----------------------------------------------------------------
                                condition |
                        DrinkWise Control |   .4204646    .189032    -1.93   0.054     .1741996    1.014873
                                Text-Only |   1.221353   .4458282     0.55   0.584     .5972123    2.497777
                         Text + Pictogram |   1.317054   .5038209     0.72   0.472     .6222832    2.787527
                         Text + Photograph |   .5924529   .2553734    -1.21   0.225     .2545355    1.378984
                                          |
                                RET_sum_b |
                      High (6-8 exposures) |    .665623   .2470517    -1.10   0.273     .3215864    1.377714
                                          |
                      condition#RET_sum_b |
   DrinkWise Control#High (6-8 exposures) |   2.154063   1.211465     1.36   0.172      .715377    6.486073
           Text-Only#High (6-8 exposures) |   .8866423   .4394927    -0.24   0.808     .3356009    2.342469
    Text + Pictogram#High (6-8 exposures) |   .8231869   .4181744    -0.38   0.702     .3041535    2.227943
   Text + Photograph#High (6-8 exposures) |   2.249363   1.208419     1.51   0.131     .7848216    6.446858
                                          |
                          Quota_Age_Group |
                                    30-49 |   .6435974   .1455802    -1.95   0.051     .4131183    1.002661
                                    50-69 |   .4924863   .1207436    -2.89   0.004     .3045817    .7963143
                                          |
                               gender_A2b |
                                 Not male |    .808233   .1497916    -1.15   0.251     .5620586    1.162229
                                          |
                                 educ_G2b |
                       Tertiary education |    .700324   .1356264    -1.84   0.066     .4791299    1.023634
                                          |
                               parent_G3r |
                                      Yes |   1.280795   .2323642     1.36   0.173     .8975389    1.827706
                                          |
                             seifa_quin_b |
                                  41-100% |   .8865497   .1533711    -0.70   0.486     .6316073    1.244397
                                          |
                              NHMRCrisk_b |
                High either STH LTH or Both |   1.511407   .2940235     2.12   0.034     1.032267    2.212947
                                          |
                                    B17_r |
Definitely/Probably drink more than should |   1.071775   .1891664     0.39   0.695     .7583476    1.514744
                                          |
                                     C1_b |
                                     Wine |   .9602506    .196759    -0.20   0.843     .6426436    1.434825
                                  Spirits |   .6249684   .1356755    -2.17   0.030     .4083845    .9564161
                                          |
                                base_7day |   .9930045   .0056406    -1.24   0.217     .9820105    1.004122
                                    _cons |   .5251909   .2087073    -1.62   0.105     .2410213    1.144403
-----------------------------------------------------------------------------------------------------------
Note: _cons estimates baseline odds.

.
.         **** Omnibus test for interaction
.         testparm i.condition#i.RET_sum_b

 ( 1)  [show_talk]2.condition#1.RET_sum_b = 0
 ( 2)  [show_talk]3.condition#1.RET_sum_b = 0
 ( 3)  [show_talk]4.condition#1.RET_sum_b = 0
 ( 4)  [show_talk]5.condition#1.RET_sum_b = 0

           chi2(  4) =    6.73
         Prob > chi2 =    0.1509
```

```
. ****************************************
. /* TABLES 3 AND 4: PAIRWISE COMPARISONS */
. *****************************************
.
. /*Significant ombibus tests for:
>                Baseline
>        D2_D4_4_r              // Intentions to avoid drinking alcohol completely in the next month (Immediately Post-Exposure)
>
>                Follow-up
>        FC1_r                  // Intentions to drink less in the next week
>
>        FC2_FC4_3_r            // Intentions to avoid drinking alcohol completely in the next month
>
>        FD2_FD6_1_b            // Awareness of alcohol-related harms: Increase your risk of cancer
>
>        neg_ar                 // Negative emotional arousal
>        pos_ar                 // Positive emotional arousal
>
> Significant interaction test (high dose v low dose of RET) for:
>        neg_ar                 // Negative emotional arousal
>
> */

. /*
>        IMPORTANT NOTE REGARDING P-VALUES
>
>        p-values for pairwise comparisons have been adjusted using the Bonferroni-Holm method for multiple comparisons
>        and d = Cohen’s d effect size. These have been calculated in excel, separately to Stata and Stata output.
>
>        */
.
```

Table 3. Pairwise comparisons for intention and knowledge outcomes for which there was a significant omnibus test

```
. di "Intentions to avoid drinking alcohol completely in the next month (Immediately Post-Exposure)"
Intentions to avoid drinking alcohol completely in the next month (Immediately Post-Exposure)

.
.         logistic D2_D4_4_r i.condition ///
>                 $covariates, ///
>                 vce(robust)

Logistic regression                             Number of obs     =      1,754
                                                Wald chi2(15)     =      70.42
                                                Prob > chi2       =     0.0000
Log pseudolikelihood = -822.27276               Pseudo R2         =     0.0467

--------------------------------------------------------------------------------------------------------------
                                             |               Robust
                                   D2_D4_4_r | Odds Ratio   Std. Err.      z    P>|z|     [95% Conf. Interval]
---------------------------------------------+----------------------------------------------------------------
                                   condition |
                           DrinkWise Control |   1.217801   .2528911     0.95   0.343     .8106142    1.829526
                                   Text-Only |   1.248006   .2559247     1.08   0.280      .834956    1.865391
                            Text + Pictogram |   1.851557   .3685619     3.09   0.002     1.253433    2.735099
                          Text + Photograph  |   1.645378   .3339455     2.45   0.014     1.105366    2.449207
                                             |
                             Quota_Age_Group |
                                       30-49 |   .8198903     .13801    -1.18   0.238     .5894883    1.140345
                                       50-69 |   .5057728   .1032261    -3.34   0.001     .3390228    .7545398
                                             |
                                   gender_A2b |
                                    Not male |   .8289013   .1231927    -1.26   0.207     .6194351      1.1092
                                             |
                                    educ_G2b |
                          Tertiary education |    1.33647   .2058241     1.88   0.060     .9882554    1.807379
                                             |
                                   parent_G3r |
                                         Yes |   1.238765   .1840739     1.44   0.150     .9257737    1.657574
                                             |
                                seifa_quin_b |
                                     41-100% |   .6911589   .0925766    -2.76   0.006     .5315751    .8986514
                                             |
                                 NHMRCrisk_b |
                 High either STH LTH or Both |   .7972699   .1197992    -1.51   0.132     .5938849    1.070307
                                             |
                                       B17_r |
     Definitely/Probably drink more than should |   1.865468   .2587117     4.50   0.000     1.421475     2.44814
                                             |
                                        C1_b |
                                        Wine |   .9146437   .1509602    -0.54   0.589     .6618548    1.263983
                                     Spirits |   .6868639   .1149102    -2.25   0.025     .4948429    .9533975
                                             |
                                   base_7day |    .972305   .0073929    -3.69   0.000     .9579226    .9869033
                                       _cons |   .2998694   .0806247    -4.48   0.000     .1770406    .5079153
--------------------------------------------------------------------------------------------------------------
Note: _cons estimates baseline odds.

.
.         pwcompare i.condition, effects asbal or

Pairwise comparisons of marginal linear predictions

Margins      : asbalanced

-------------------------------------------------------------------------------------------------------------------------
                                                        |                           Unadjusted           Unadjusted
                                                        | Odds Ratio   Std. Err.       z    P>|z|     [95% Conf. Interval]
--------------------------------------------------------+----------------------------------------------------------------
D2_D4_4_r                                               |
                                              condition |
  DrinkWise Control vs No Health Warning Label Control  |   1.217801   .2528911     0.95   0.343     .8106142    1.829526
          Text-Only vs No Health Warning Label Control  |   1.248006   .2559247     1.08   0.280      .834956    1.865391
   Text + Pictogram vs No Health Warning Label Control  |   1.851557   .3685619     3.09   0.002     1.253433    2.735099
  Text + Photograph vs No Health Warning Label Control  |   1.645378   .3339455     2.45   0.014     1.105366    2.449207
                        Text-Only vs DrinkWise Control  |   1.024803   .2032065     0.12   0.902     .6947954    1.511554
                 Text + Pictogram vs DrinkWise Control  |   1.520409    .292557     2.18   0.029     1.042731    2.216913
                Text + Photograph vs DrinkWise Control  |   1.351106     .26501     1.53   0.125     .9198819    1.984479
                         Text + Pictogram vs Text-Only  |   1.483612   .2810327     2.08   0.037     1.023485    2.150596
                        Text + Photograph vs Text-Only  |   1.318406    .253553     1.44   0.151     .9043732    1.921986
                 Text + Photograph vs Text + Pictogram  |    .888646    .166407    -0.63   0.528     .6156496    1.282697
-------------------------------------------------------------------------------------------------------------------------
```

```
. di "Intentions to drink less in the next week  (FOLLOW-UP)"
Intentions to drink less in the next week  (FOLLOW-UP)

.
.         logistic FC1_r i.condition ///
>                 $covariates     ///
>                 if FUP_compl == 1,      ///
>                 vce(robust)

Logistic regression                             Number of obs     =      1,086
                                                Wald chi2(15)     =      47.13
                                                Prob > chi2       =     0.0000
Log pseudolikelihood = -726.99946               Pseudo R2         =     0.0335

------------------------------------------------------------------------------------------------------------
                                            |               Robust
                                      FC1_r | Odds Ratio   Std. Err.      z    P>|z|     [95% Conf. Interval]
--------------------------------------------+---------------------------------------------------------------
                                  condition |
                          DrinkWise Control |   1.140287   .2296119     0.65   0.514     .7684463    1.692057
                                  Text-Only |   1.389915   .2752216     1.66   0.096     .9428425    2.048979
                           Text + Pictogram |   2.004214    .405316     3.44   0.001     1.348354    2.979094
                          Text + Photograph |   1.371456   .2773107     1.56   0.118     .9227147    2.038432
                                            |
                            Quota_Age_Group |
                                      30-49 |   1.072511   .1747434     0.43   0.667     .7793202    1.476003
                                      50-69 |   .7463149   .1307353    -1.67   0.095     .5294359    1.052037
                                            |
                                 gender_A2b |
                                   Not male |   1.090011   .1577102     0.60   0.551     .8208677      1.4474
                                            |
                                   educ_G2b |
                         Tertiary education |   1.246161    .193202     1.42   0.156     .9196141    1.688661
                                            |
                                 parent_G3r |
                                        Yes |   1.107307   .1530533     0.74   0.461      .844528    1.451852
                                            |
                               seifa_quin_b |
                                    41-100% |   .9623781   .1328468    -0.28   0.781     .7342531    1.261379
                                            |
                                NHMRCrisk_b |
                  High either STH LTH or Both |   .7914095   .1203112    -1.54   0.124       .58749     1.06611
                                            |
                                      B17_r |
Definitely/Probably drink more than should |   2.146418   .3126398     5.24   0.000      1.61336      2.8556
                                            |
                                       C1_b |
                                       Wine |   .9006297   .1494895    -0.63   0.528     .6505205      1.2469
                                    Spirits |   .8478167   .1407535    -0.99   0.320     .6123313    1.173863
                                            |
                                  base_7day |   .9906747   .0046404    -2.00   0.045     .9816214    .9998116
                                      _cons |   .5822294   .1618321    -1.95   0.052     .3376763    1.003894
------------------------------------------------------------------------------------------------------------
Note: _cons estimates baseline odds.

.
.         pwcompare i.condition, eff asbal or

Pairwise comparisons of marginal linear predictions

Margins      : asbalanced

-----------------------------------------------------------------------------------------------------------------------
                                                       |                            Unadjusted           Unadjusted
                                                       | Odds Ratio   Std. Err.      z    P>|z|     [95% Conf. Interval]
-------------------------------------------------------+---------------------------------------------------------------
FC1_r                                                  |
                                             condition |
DrinkWise Control vs No Health Warning Label Control   |   1.140287   .2296119     0.65   0.514     .7684463    1.692057
        Text-Only vs No Health Warning Label Control   |   1.389915   .2752216     1.66   0.096     .9428425    2.048979
 Text + Pictogram vs No Health Warning Label Control   |   2.004214    .405316     3.44   0.001     1.348354    2.979094
Text + Photograph vs No Health Warning Label Control   |   1.371456   .2773107     1.56   0.118     .9227147    2.038432
                       Text-Only vs DrinkWise Control  |   1.218917   .2361984     1.02   0.307     .8337398    1.782041
                Text + Pictogram vs DrinkWise Control  |   1.757639   .3481372     2.85   0.004     1.192152    2.591362
               Text + Photograph vs DrinkWise Control  |   1.202729   .2358205     0.94   0.346     .8189765    1.766297
                        Text + Pictogram vs Text-Only  |   1.441968   .2812154     1.88   0.061     .9839041    2.113288
                       Text + Photograph vs Text-Only  |   .9867191   .1915423    -0.07   0.945     .6744627    1.443541
                Text + Photograph vs Text + Pictogram  |   .6842863   .1353373    -1.92   0.055     .4643957    1.008295
-----------------------------------------------------------------------------------------------------------------------
```

```
. di "Intentions to avoid drinking alcohol completely in the next month (FOLLOW-UP)"
Intentions to avoid drinking alcohol completely in the next month (FOLLOW-UP)

.
.         logistic FC2_FC4_3_r i.condition ///
>                 $covariates     ///
>                 if FUP_compl == 1,      ///
>                 vce(robust)

Logistic regression                             Number of obs     =      1,086
                                                Wald chi2(15)     =      43.90
                                                Prob > chi2       =     0.0001
Log pseudolikelihood = -461.01096               Pseudo R2         =     0.0547

-------------------------------------------------------------------------------------------------------------
                                             |               Robust
                                 FC2_FC4_3_r | Odds Ratio   Std. Err.      z    P>|z|     [95% Conf. Interval]
---------------------------------------------+---------------------------------------------------------------
                                   condition |
                          DrinkWise Control  |   .6016474   .1809486    -1.69   0.091     .3336866    1.084789
                                  Text-Only  |   1.208902    .316214     0.73   0.468     .7240044    2.018556
                           Text + Pictogram  |   1.557418   .4021538     1.72   0.086     .9388786    2.583456
                          Text + Photograph  |   1.324193   .3461137     1.07   0.283     .7933531    2.210222
                                             |
                             Quota_Age_Group |
                                      30-49  |   .5609676   .1290159    -2.51   0.012     .3574139    .8804488
                                      50-69  |   .3536873   .0946068    -3.89   0.000     .2093794    .5974548
                                             |
                                  gender_A2b |
                                   Not male  |   .6333794   .1294655    -2.23   0.025     .4243016     .945482
                                             |
                                    educ_G2b |
                         Tertiary education  |   1.036006   .2266213     0.16   0.872      .674786    1.590591
                                             |
                                  parent_G3r |
                                        Yes  |   1.455168   .2903058     1.88   0.060     .9842348    2.151433
                                             |
                                seifa_quin_b |
                                    41-100%  |   .8254059   .1507738    -1.05   0.294     .5770079    1.180738
                                             |
                                  NHMRCrisk_b |
                  High either STH LTH or Both |   .7565258    .160694    -1.31   0.189      .498907     1.14717
                                             |
                                       B17_r |
Definitely/Probably drink more than should   |   1.466903   .2852146     1.97   0.049     1.002073    2.147352
                                             |
                                        C1_b |
                                       Wine  |   .8959072    .198497    -0.50   0.620     .5803246    1.383105
                                    Spirits  |   .6844941   .1543583    -1.68   0.093     .4399645    1.064932
                                             |
                                   base_7day |    .979048   .0107087    -1.94   0.053     .9582827    1.000263
                                       _cons |   .4615474   .1711203    -2.09   0.037     .2231671     .954558
-------------------------------------------------------------------------------------------------------------
Note: _cons estimates baseline odds.

.
.         pwcompare i.condition, eff asbal or

Pairwise comparisons of marginal linear predictions

Margins      : asbalanced

-------------------------------------------------------------------------------------------------------------------------
                                                         |                            Unadjusted           Unadjusted
                                                         | Odds Ratio   Std. Err.      z    P>|z|     [95% Conf. Interval]
---------------------------------------------------------+---------------------------------------------------------------
FC2_FC4_3_r                                              |
                                               condition |
DrinkWise Control vs No Health Warning Label Control     |   .6016474   .1809486    -1.69   0.091     .3336866    1.084789
        Text-Only vs No Health Warning Label Control     |   1.208902    .316214     0.73   0.468     .7240044    2.018556
 Text + Pictogram vs No Health Warning Label Control     |   1.557418   .4021538     1.72   0.086     .9388786    2.583456
Text + Photograph vs No Health Warning Label Control     |   1.324193   .3461137     1.07   0.283     .7933531    2.210222
                      Text-Only vs DrinkWise Control     |    2.00932   .5838028     2.40   0.016     1.136932    3.551105
               Text + Pictogram vs DrinkWise Control     |   2.588589   .7478721     3.29   0.001     1.469405    4.560209
              Text + Photograph vs DrinkWise Control     |   2.200945   .6453792     2.69   0.007     1.238833    3.910258
                       Text + Pictogram vs Text-Only     |   1.288292   .3179295     1.03   0.305     .7942393    2.089666
                      Text + Photograph vs Text-Only     |   1.095368    .274254     0.36   0.716      .670563     1.78929
               Text + Photograph vs Text + Pictogram     |   .8502486   .2126396    -0.65   0.517     .5207964     1.38811
-------------------------------------------------------------------------------------------------------------------------
```

```
. di "Awareness of alcohol-related harms: Increase your risk of cancer (FOLLOW-UP)"
Awareness of alcohol-related harms: Increase your risk of cancer (FOLLOW-UP)

. 
.         logistic FD2_FD6_1_b i.condition ///
>                 $covariates     ///
>                 if FUP_compl == 1,      ///
>                 vce(robust)

Logistic regression                             Number of obs     =      1,086
                                                Wald chi2(15)     =      36.11
                                                Prob > chi2       =     0.0017
Log pseudolikelihood = -676.75525               Pseudo R2         =     0.0286

------------------------------------------------------------------------------------------------------------
                                            |               Robust
                                FD2_FD6_1_b | Odds Ratio   Std. Err.      z    P>|z|     [95% Conf. Interval]
--------------------------------------------+---------------------------------------------------------------
                                  condition |
                         DrinkWise Control  |   .8190231   .1640185    -1.00   0.319     .5531381    1.212715
                                 Text-Only  |   1.268224   .2633384     1.14   0.252     .8442081    1.905208
                          Text + Pictogram  |   1.325797    .281197     1.33   0.184     .8748634    2.009158
                         Text + Photograph  |   1.372612   .2835643     1.53   0.125     .9155882    2.057762
                                            |
                            Quota_Age_Group |
                                     30-49  |   .8988074   .1521756    -0.63   0.529     .6449872    1.252513
                                     50-69  |   .6707413   .1214409    -2.21   0.027     .4703714    .9564652
                                            |
                                 gender_A2b |
                                  Not male  |   1.025124   .1548021     0.16   0.869     .7624972    1.378208
                                            |
                                   educ_G2b |
                        Tertiary education  |    .905637   .1480209    -0.61   0.544      .657401    1.247607
                                            |
                                 parent_G3r |
                                       Yes  |   1.120137   .1602703     0.79   0.428     .8462146     1.48273
                                            |
                               seifa_quin_b |
                                   41-100%  |   1.313772   .1876771     1.91   0.056     .9929397     1.73827
                                            |
                                 NHMRCrisk_b |
                 High either STH LTH or Both |   .8109323   .1278597    -1.33   0.184     .5953545    1.104571
                                            |
                                      B17_r |
Definitely/Probably drink more than should  |   1.846489   .2836048     3.99   0.000       1.3665    2.495077
                                            |
                                       C1_b |
                                      Wine  |   1.125629   .1962003     0.68   0.497     .7998867    1.584025
                                   Spirits  |   .8051101   .1365393    -1.28   0.201       .57743    1.122564
                                            |
                                  base_7day |   .9957386   .0049611    -0.86   0.391     .9860624     1.00551
                                      _cons |   1.537102   .4425806     1.49   0.135     .8742081    2.702656
------------------------------------------------------------------------------------------------------------
Note: _cons estimates baseline odds.

. 
.         pwcompare i.condition, eff asbal or

Pairwise comparisons of marginal linear predictions

Margins      : asbalanced

-----------------------------------------------------------------------------------------------------------------------
                                                       |                           Unadjusted           Unadjusted
                                                       | Odds Ratio   Std. Err.      z    P>|z|     [95% Conf. Interval]
-------------------------------------------------------+---------------------------------------------------------------
FD2_FD6_1_b                                            |
                                             condition |
DrinkWise Control vs No Health Warning Label Control   |   .8190231   .1640185    -1.00   0.319     .5531381    1.212715
        Text-Only vs No Health Warning Label Control   |   1.268224   .2633384     1.14   0.252     .8442081    1.905208
 Text + Pictogram vs No Health Warning Label Control   |   1.325797    .281197     1.33   0.184     .8748634    2.009158
Text + Photograph vs No Health Warning Label Control   |   1.372612   .2835643     1.53   0.125     .9155882    2.057762
                      Text-Only vs DrinkWise Control   |   1.548459    .315636     2.15   0.032     1.038465    2.308913
               Text + Pictogram vs DrinkWise Control   |   1.618754   .3360533     2.32   0.020     1.077635     2.43159
              Text + Photograph vs DrinkWise Control   |   1.675913   .3365963     2.57   0.010     1.130559    2.484333
                       Text + Pictogram vs Text-Only   |   1.045397   .2253888     0.21   0.837     .6851113    1.595149
                      Text + Photograph vs Text-Only   |    1.08231   .2269324     0.38   0.706     .7175908      1.6324
               Text + Photograph vs Text + Pictogram   |    1.03531   .2210644     0.16   0.871      .681268    1.573342
-----------------------------------------------------------------------------------------------------------------------
```

Table 4. Pairwise comparisons for negative emotional arousal and positive emotional arousal

```
. di "Negative emotional arousal (FOLLOW-UP)"
Negative emotional arousal (FOLLOW-UP)

.
.         regress neg_ar i.condition ///
>                 $covariates     ///
>                 if FUP_compl == 1,      ///
>                 vce(hc3)

Linear regression                               Number of obs     =      1,086
                                                F(15, 1070)       =      22.29
                                                Prob > F          =     0.0000
                                                R-squared         =     0.1982
                                                Root MSE          =     1.3849

--------------------------------------------------------------------------------------------------------------
                                             |             Robust HC3
                                      neg_ar |      Coef.   Std. Err.      t    P>|t|     [95% Conf. Interval]
---------------------------------------------+----------------------------------------------------------------
                                   condition |
                           DrinkWise Control |  -.0172355   .1183804    -0.15   0.884    -.2495195    .2150484
                                   Text-Only |   .9278281   .1352043     6.86   0.000     .6625325    1.193124
                            Text + Pictogram |   1.167013   .1359341     8.59   0.000     .9002848     1.43374
                           Text + Photograph |   1.332357   .1366632     9.75   0.000     1.064199    1.600516
                                             |
                             Quota_Age_Group |
                                       30-49 |  -.3970216   .1188105    -3.34   0.001    -.6301497   -.1638935
                                       50-69 |  -.8279297   .1214112    -6.82   0.000    -1.066161   -.5896987
                                             |
                                  gender_A2b |
                                    Not male |  -.1418477   .1016601    -1.40   0.163    -.3413234    .0576281
                                             |
                                    educ_G2b |
                          Tertiary education |  -.0579472   .1057635    -0.55   0.584    -.2654746    .1495802
                                             |
                                  parent_G3r |
                                         Yes |   .1533314   .0986591     1.55   0.120    -.0402558    .3469186
                                             |
                                seifa_quin_b |
                                     41-100% |   .1323073   .0915119     1.45   0.149    -.0472559    .3118705
                                             |
                                 NHMRCrisk_b |
                   High either STH LTH or Both |  -.2087698   .1027051    -2.03   0.042    -.4102962   -.0072435
                                             |
                                       B17_r |
      Definitely/Probably drink more than should |   .1489471   .0964864     1.54   0.123     -.040377    .3382711
                                             |
                                        C1_b |
                                        Wine |   .0684551   .1171794     0.58   0.559    -.1614724    .2983826
                                     Spirits |  -.4563884   .1095617    -4.17   0.000    -.6713685   -.2414083
                                             |
                                   base_7day |  -.0010737   .0030774    -0.35   0.727    -.0071121    .0049648
                                       _cons |   2.310982   .1913675    12.08   0.000     1.935484     2.68648
--------------------------------------------------------------------------------------------------------------

.
.         margins i.condition, pwcompare(effects) asbal atmeans

Pairwise comparisons of adjusted predictions    Number of obs     =      1,086
Model VCE    : Robust HC3

Expression   : Linear prediction, predict()
at           : condition                    (asbalanced)
               Quota_Age_Group              (asbalanced)
               gender_A2b                   (asbalanced)
               educ_G2b                     (asbalanced)
               parent_G3r                   (asbalanced)
               seifa_quin_b                 (asbalanced)
               NHMRCrisk_b                  (asbalanced)
               B17_r                        (asbalanced)
               C1_b                         (asbalanced)
               base_7day       =    14.63444 (mean)

------------------------------------------------------------------------------------------------------------------------
                                                       |            Delta-method    Unadjusted           Unadjusted
                                                       |   Contrast   Std. Err.      t    P>|t|     [95% Conf. Interval]
-------------------------------------------------------+----------------------------------------------------------------
                                             condition |
DrinkWise Control vs No Health Warning Label Control |  -.0172355   .1183804    -0.15   0.884    -.2495195    .2150484
        Text-Only vs No Health Warning Label Control |   .9278281   .1352043     6.86   0.000     .6625325    1.193124
 Text + Pictogram vs No Health Warning Label Control |   1.167013   .1359341     8.59   0.000     .9002848     1.43374
Text + Photograph vs No Health Warning Label Control |   1.332357   .1366632     9.75   0.000     1.064199    1.600516
                       Text-Only vs DrinkWise Control |   .9450636   .1263326     7.48   0.000      .697176    1.192951
                Text + Pictogram vs DrinkWise Control |   1.184248   .1282805     9.23   0.000     .9325383    1.435958
               Text + Photograph vs DrinkWise Control |   1.349593   .1288024    10.48   0.000     1.096859    1.602327
                        Text + Pictogram vs Text-Only |   .2391845   .1432165     1.67   0.095    -.0418326    .5202015
                       Text + Photograph vs Text-Only |   .4045294   .1437789     2.81   0.005     .1224088      .68665
                Text + Photograph vs Text + Pictogram |   .1653449   .1456228     1.14   0.256    -.1203937    .4510836
------------------------------------------------------------------------------------------------------------------------

.
.
.                 *** Cohen's d -----
.                 qui {

.
.                 *Show Cohen's d
.                 di "Cohen's d Effect size:"
Cohen's d Effect size:

.                 di "`var'"


.                 mat l cont_mat_d

cont_mat_d[10,1]
              d
 r1  -.00890192
 r2   .41958045
 r3   .52491034
 r4   .59608384
 r5   .45738706
 r6   .56444329
 r7   .64064438
 r8   .10211228
 r9    .1720256
r10   .06942241
```

```
.         ** Interaction
.         regress neg_ar i.condition##i.RET_sum_b ///
>                 $covariates     ///
>                 if FUP_compl == 1,      ///
>                 vce(hc3)

Linear regression                               Number of obs     =      1,086
                                                F(20, 1065)       =      18.91
                                                Prob > F          =     0.0000
                                                R-squared         =     0.2091
                                                Root MSE          =     1.3787

------------------------------------------------------------------------------------------------------------
                                             |             Robust HC3
                                      neg_ar |      Coef.   Std. Err.      t    P>|t|     [95% Conf. Interval]
---------------------------------------------+--------------------------------------------------------------
                                   condition |
                           DrinkWise Control  |  -.0106624   .2267684    -0.05   0.963     -.455626    .4343011
                                   Text-Only  |   .5772558   .2460482     2.35   0.019     .0944615     1.06005
                            Text + Pictogram  |   .8132995    .253801     3.20   0.001     .3152928    1.311306
                          Text + Photograph   |   .8234354   .2519444     3.27   0.001     .3290716    1.317799
                                              |
                                   RET_sum_b  |
                         High (6-8 exposures) |  -.4736515    .201101    -2.36   0.019    -.8682507   -.0790524
                                              |
                         condition#RET_sum_b  |
       DrinkWise Control#High (6-8 exposures) |   .0011617   .2626874     0.00   0.996     -.514282    .5166053
               Text-Only#High (6-8 exposures) |   .5656017   .2910462     1.94   0.052    -.0054873    1.136691
        Text + Pictogram#High (6-8 exposures) |    .565725   .2985532     1.89   0.058    -.0200942    1.151544
       Text + Photograph#High (6-8 exposures) |   .7925111   .2975524     2.66   0.008     .2086555    1.376367
                                              |
                             Quota_Age_Group  |
                                       30-49  |  -.3753123    .120055    -3.13   0.002    -.6108835   -.1397412
                                       50-69  |  -.8060134   .1217867    -6.62   0.000    -1.044983   -.5670443
                                              |
                                  gender_A2b  |
                                    Not male  |  -.1428926   .1012051    -1.41   0.158    -.3414767    .0556915
                                              |
                                    educ_G2b  |
                          Tertiary education  |  -.0554779   .1052572    -0.53   0.598     -.262013    .1510572
                                              |
                                  parent_G3r  |
                                         Yes  |   .1477909   .0990254     1.49   0.136    -.0465162     .342098
                                              |
                                seifa_quin_b  |
                                     41-100%  |   .1235781   .0912511     1.35   0.176    -.0554742    .3026304
                                              |
                                  NHMRCrisk_b |
                   High either STH LTH or Both |  -.2084985   .1020555    -2.04   0.041    -.4087512   -.0082459
                                              |
                                       B17_r  |
       Definitely/Probably drink more than should |   .1458654   .0956687     1.52   0.128    -.0418552     .333586
                                              |
                                        C1_b  |
                                        Wine  |   .0836195   .1166802     0.72   0.474    -.1453296    .3125686
                                     Spirits  |  -.4492333   .1093574    -4.11   0.000    -.6638137    -.234653
                                              |
                                   base_7day  |  -.0012111   .0029948    -0.40   0.686    -.0070875    .0046653
                                       _cons  |   2.596674   .2466687    10.53   0.000     2.112663    3.080686
------------------------------------------------------------------------------------------------------------

.
.         *LOW
.         margins i.condition#i(0).RET_sum_b, pwcompare(effects) asbal atmeans

Pairwise comparisons of adjusted predictions    Number of obs     =      1,086
Model VCE    : Robust HC3

Expression   : Linear prediction, predict()
at           : condition                     (asbalanced)
               RET_sum_b                     (asbalanced)
               Quota_Age_Group               (asbalanced)
               gender_A2b                    (asbalanced)
               educ_G2b                      (asbalanced)
               parent_G3r                    (asbalanced)
               seifa_quin_b                  (asbalanced)
               NHMRCrisk_b                   (asbalanced)
               B17_r                         (asbalanced)
               C1_b                          (asbalanced)
               base_7day       =    14.63444 (mean)

--------------------------------------------------------------------------------------------------------------------------------
                                                                  |            Delta-method    Unadjusted           Unadjusted
                                                                  |   Contrast   Std. Err.      t    P>|t|     [95% Conf. Interval]
------------------------------------------------------------------+-------------------------------------------------------------
                                              condition#RET_sum_b |
                          (DrinkWise Control#Low (0-5 exposures)) |
                                                               vs |
               (No Health Warning Label Control#Low (0-5 exposures)) |  -.0106624   .2267684    -0.05   0.963     -.455626    .4343011
                                  (Text-Only#Low (0-5 exposures)) |
                                                               vs |
               (No Health Warning Label Control#Low (0-5 exposures)) |   .5772558   .2460482     2.35   0.019     .0944615     1.06005
                           (Text + Pictogram#Low (0-5 exposures)) |
                                                               vs |
               (No Health Warning Label Control#Low (0-5 exposures)) |   .8132995    .253801     3.20   0.001     .3152928    1.311306
                          (Text + Photograph#Low (0-5 exposures)) |
                                                               vs |
               (No Health Warning Label Control#Low (0-5 exposures)) |   .8234354   .2519444     3.27   0.001     .3290716    1.317799
 (Text-Only#Low (0-5 exposures)) vs (DrinkWise Control#Low (0-5 exposures)) |   .5879182   .2248267     2.61   0.009     .1467646    1.029072
                           (Text + Pictogram#Low (0-5 exposures)) |
                                                               vs |
                          (DrinkWise Control#Low (0-5 exposures)) |    .823962   .2335777     3.53   0.000     .3656373    1.282287
                          (Text + Photograph#Low (0-5 exposures)) |
                                                               vs |
                          (DrinkWise Control#Low (0-5 exposures)) |   .8340978   .2314042     3.60   0.000     .3800379    1.288158
 (Text + Pictogram#Low (0-5 exposures)) vs (Text-Only#Low (0-5 exposures)) |   .2360438   .2517348     0.94   0.349    -.2579086    .7299962
(Text + Photograph#Low (0-5 exposures)) vs (Text-Only#Low (0-5 exposures)) |   .2461796    .250843     0.98   0.327    -.2460231    .7383823
                          (Text + Photograph#Low (0-5 exposures)) |
                                                               vs |
                           (Text + Pictogram#Low (0-5 exposures)) |   .0101358   .2587986     0.04   0.969    -.4976772    .5179489
--------------------------------------------------------------------------------------------------------------------------------

.
.
.                 *** Cohen's d ----
.                 * Loop through rows
.                 qui {

.                 di "-- Cohen's d effect size --"
-- Cohen's d effect size --

.                 mat l int_low_d

int_low_d[10,1]
             d
 r1  -.00288157
 r2   .14378161
 r3   .19638688
 r4   .20029957
 r5   .16025966
 r6   .21618773
 r7   .22090265
 r8   .05746515
 r9   .06014579
r10   .00240023
```

```
.         *HIGH
.         margins i.condition#i(1).RET_sum_b, pwcompare(effects) asbal atmeans

Pairwise comparisons of adjusted predictions    Number of obs     =      1,086
Model VCE    : Robust HC3

Expression   : Linear prediction, predict()
at           : condition                     (asbalanced)
               RET_sum_b                     (asbalanced)
               Quota_Age_Group               (asbalanced)
               gender_A2b                    (asbalanced)
               educ_G2b                      (asbalanced)
               parent_G3r                    (asbalanced)
               seifa_quin_b                  (asbalanced)
               NHMRCrisk_b                   (asbalanced)
               B17_r                         (asbalanced)
               C1_b                          (asbalanced)
               base_7day       =    14.63444 (mean)

------------------------------------------------------------------------------------------------------------------------------------------------------
                                                                              |            Delta-method    Unadjusted           Unadjusted
                                                                              |   Contrast   Std. Err.      t    P>|t|     [95% Conf. Interval]
------------------------------------------------------------------------------+----------------------------------------------------------------------
                                                            condition#RET_sum_b |
                                        (DrinkWise Control#High (6-8 exposures)) |
                                                                              vs |
                     (No Health Warning Label Control#High (6-8 exposures)) |  -.0095008   .1304137    -0.07   0.942    -.2653978    .2463963
                                                (Text-Only#High (6-8 exposures)) |
                                                                              vs |
                     (No Health Warning Label Control#High (6-8 exposures)) |   1.142858    .156138     7.32   0.000     .8364845    1.449231
                                         (Text + Pictogram#High (6-8 exposures)) |
                                                                              vs |
                     (No Health Warning Label Control#High (6-8 exposures)) |   1.379025   .1558613     8.85   0.000     1.073194    1.684855
                                        (Text + Photograph#High (6-8 exposures)) |
                                                                              vs |
                     (No Health Warning Label Control#High (6-8 exposures)) |   1.615946   .1579679    10.23   0.000     1.305983     1.92591
(Text-Only#High (6-8 exposures)) vs (DrinkWise Control#High (6-8 exposures)) |   1.152358   .1499275     7.69   0.000     .8581713    1.446545
                                         (Text + Pictogram#High (6-8 exposures)) |
                                                                              vs |
                                        (DrinkWise Control#High (6-8 exposures)) |   1.388525   .1505276     9.22   0.000     1.093161     1.68389
                                        (Text + Photograph#High (6-8 exposures)) |
                                                                              vs |
                                        (DrinkWise Control#High (6-8 exposures)) |   1.625447    .151628    10.72   0.000     1.327924    1.922971
 (Text + Pictogram#High (6-8 exposures)) vs (Text-Only#High (6-8 exposures)) |    .236167   .1732172     1.36   0.173    -.1037187    .5760527
(Text + Photograph#High (6-8 exposures)) vs (Text-Only#High (6-8 exposures)) |   .4730889   .1738794     2.72   0.007     .1319038     .814274
                                        (Text + Photograph#High (6-8 exposures)) |
                                                                              vs |
                                         (Text + Pictogram#High (6-8 exposures)) |   .2369219   .1745666     1.36   0.175    -.1056116    .5794554
------------------------------------------------------------------------------------------------------------------------------------------------------

.
.
.                *** Cohen's d ----
.                qui {

.                di "-- Cohen's d effect size --"
-- Cohen's d effect size --

.                mat l int_high_d

int_high_d[10,1]
              d
 r1  -.00446468
 r2   .44857869
 r3   .54223653
 r4   .62692176
 r5   .47104379
 r6   .56531794
 r7   .65697451
 r8   .08355713
 r9   .16674387
r10   .08317623
```

```
. di "Positive emotional arousal (FOLLOW-UP)"
Positive emotional arousal (FOLLOW-UP)

.
.         regress pos_ar i.condition ///
>                 $covariates     ///
>                 if FUP_compl == 1,      ///
>                 vce(hc3)

Linear regression                               Number of obs     =      1,086
                                                F(15, 1070)       =       9.68
                                                Prob > F          =     0.0000
                                                R-squared         =     0.1124
                                                Root MSE          =     1.4743

--------------------------------------------------------------------------------------------------------------
                                             |               Robust HC3
                                      pos_ar |      Coef.   Std. Err.      t    P>|t|     [95% Conf. Interval]
---------------------------------------------+----------------------------------------------------------------
                                   condition |
                           DrinkWise Control |  -.2131562   .1463552    -1.46   0.146     -.500332    .0740197
                                   Text-Only |  -.5465061    .144575    -3.78   0.000    -.8301888   -.2628233
                            Text + Pictogram |  -.4019307   .1410994    -2.85   0.004    -.6787936   -.1250679
                           Text + Photograph |  -.7208618   .1494939    -4.82   0.000    -1.014196   -.4275274
                                             |
                             Quota_Age_Group |
                                       30-49 |  -.4426639   .1255961    -3.52   0.000    -.6891065   -.1962214
                                       50-69 |  -.9990384   .1284695    -7.78   0.000    -1.251119   -.7469576
                                             |
                                  gender_A2b |
                                    Not male |  -.2081605   .1083726    -1.92   0.055    -.4208074    .0044865
                                             |
                                    educ_G2b |
                          Tertiary education |  -.1588226   .1137662    -1.40   0.163    -.3820527    .0644075
                                             |
                                  parent_G3r |
                                         Yes |   .0844032   .1040727     0.81   0.418    -.1198066     .288613
                                             |
                                seifa_quin_b |
                                     41-100% |  -.1001702   .1016711    -0.99   0.325    -.2996675    .0993271
                                             |
                                 NHMRCrisk_b |
                   High either STH LTH or Both |  -.0045559   .1077264    -0.04   0.966    -.2159348     .206823
                                             |
                                      B17_r |
    Definitely/Probably drink more than should |  -.0707086   .1055128    -0.67   0.503     -.277744    .1363268
                                             |
                                        C1_b |
                                        Wine |   -.458306   .1217381    -3.76   0.000    -.6971784   -.2194335
                                     Spirits |  -.4772578   .1231698    -3.87   0.000    -.7189395    -.235576
                                             |
                                   base_7day |   .0019298   .0037422     0.52   0.606     -.005413    .0092727
                                       _cons |   4.258536   .2005323    21.24   0.000     3.865054    4.652017
--------------------------------------------------------------------------------------------------------------

.
.         margins i.condition, pwcompare(effects) asbal atmeans

Pairwise comparisons of adjusted predictions    Number of obs     =      1,086
Model VCE    : Robust HC3

Expression   : Linear prediction, predict()
at           : condition                     (asbalanced)
               Quota_Age_Group               (asbalanced)
               gender_A2b                    (asbalanced)
               educ_G2b                      (asbalanced)
               parent_G3r                    (asbalanced)
               seifa_quin_b                  (asbalanced)
               NHMRCrisk_b                   (asbalanced)
               B17_r                         (asbalanced)
               C1_b                          (asbalanced)
               base_7day       =    14.63444 (mean)

-----------------------------------------------------------------------------------------------------------------------
                                                     |            Delta-method    Unadjusted           Unadjusted
                                                     |   Contrast   Std. Err.      t    P>|t|     [95% Conf. Interval]
-----------------------------------------------------+-----------------------------------------------------------------
                                           condition |
DrinkWise Control vs No Health Warning Label Control |  -.2131562   .1463552    -1.46   0.146     -.500332    .0740197
        Text-Only vs No Health Warning Label Control |  -.5465061    .144575    -3.78   0.000    -.8301888   -.2628233
 Text + Pictogram vs No Health Warning Label Control |  -.4019307   .1410994    -2.85   0.004    -.6787936   -.1250679
Text + Photograph vs No Health Warning Label Control |  -.7208618   .1494939    -4.82   0.000    -1.014196   -.4275274
                      Text-Only vs DrinkWise Control |  -.3333499   .1411681    -2.36   0.018    -.6103476   -.0563521
               Text + Pictogram vs DrinkWise Control |  -.1887746   .1379017    -1.37   0.171    -.4593631    .0818139
              Text + Photograph vs DrinkWise Control |  -.5077057    .145959    -3.48   0.001    -.7941039   -.2213074
                       Text + Pictogram vs Text-Only |   .1445753   .1362311     1.06   0.289    -.1227351    .4118857
                      Text + Photograph vs Text-Only |  -.1743558   .1454178    -1.20   0.231    -.4596921    .1109805
               Text + Photograph vs Text + Pictogram |  -.3189311    .141836    -2.25   0.025    -.5972394   -.0406228
-----------------------------------------------------------------------------------------------------------------------

.
.                         *Cohen's d -----
.                         qui {

.
.                         *Show Cohen's d
.                         di "Cohen's d Effect size:"
Cohen's d Effect size:

.                         di "`var'"


.                         mat l cont_mat_d

cont_mat_d[10,1]
              d
 r1  -.08904873
 r2  -.23112118
 r3  -.17416633
 r4  -.29482668
 r5  -.14437826
 r6  -.08369738
 r7  -.21267643
 r8   .06488674
 r9  -.07330895
r10  -.13748282
```

Fig 3. Adjusted means for negative emotional arousal by condition type and dose of repeated exposure (N = 1,087).

```
. ****************************************************************************
. /*FIGURE 3: Adjusted means for negative emotional arousal by condition type
>  and dose of repeated exposure (N = 1,087)*/
. ***************************************************************************
.         regress neg_ar i.condition##i.RET_sum_b ///
>         $covariates ///
>         if FUP_compl == 1, vce(hc3)

Linear regression                               Number of obs     =      1,086
                                                F(20, 1065)       =      18.91
                                                Prob > F          =     0.0000
                                                R-squared         =     0.2091
                                                Root MSE          =     1.3787

--------------------------------------------------------------------------------------------------------------
                                             |             Robust HC3
                                      neg_ar |      Coef.   Std. Err.      t    P>|t|     [95% Conf. Interval]
---------------------------------------------+----------------------------------------------------------------
                                   condition |
                           DrinkWise Control |  -.0106624   .2267684    -0.05   0.963     -.455626    .4343011
                                   Text-Only |   .5772558   .2460482     2.35   0.019     .0944615     1.06005
                            Text + Pictogram |   .8132995    .253801     3.20   0.001     .3152928    1.311306
                           Text + Photograph |   .8234354   .2519444     3.27   0.001     .3290716    1.317799
                                             |
                                   RET_sum_b |
                          High (6-8 exposures) |  -.4736515    .201101    -2.36   0.019    -.8682507   -.0790524
                                             |
                         condition#RET_sum_b |
     DrinkWise Control#High (6-8 exposures) |   .0011617   .2626874     0.00   0.996     -.514282    .5166053
             Text-Only#High (6-8 exposures) |   .5656017   .2910462     1.94   0.052    -.0054873    1.136691
      Text + Pictogram#High (6-8 exposures) |    .565725   .2985532     1.89   0.058    -.0200942    1.151544
     Text + Photograph#High (6-8 exposures) |   .7925111   .2975524     2.66   0.008     .2086555    1.376367
                                             |
                             Quota_Age_Group |
                                       30-49 |  -.3753123    .120055    -3.13   0.002    -.6108835   -.1397412
                                       50-69 |  -.8060134   .1217867    -6.62   0.000    -1.044983   -.5670443
                                             |
                                  gender_A2b |
                                    Not male |  -.1428926   .1012051    -1.41   0.158    -.3414767    .0556915
                                             |
                                    educ_G2b |
                          Tertiary education |  -.0554779   .1052572    -0.53   0.598     -.262013    .1510572
                                             |
                                  parent_G3r |
                                         Yes |   .1477909   .0990254     1.49   0.136    -.0465162     .342098
                                             |
                                seifa_quin_b |
                                     41-100% |   .1235781   .0912511     1.35   0.176    -.0554742    .3026304
                                             |
                                 NHMRCrisk_b |
                  High either STH LTH or Both |  -.2084985   .1020555    -2.04   0.041    -.4087512   -.0082459
                                             |
                                       B17_r |
    Definitely/Probably drink more than should |   .1458654   .0956687     1.52   0.128    -.0418552     .333586
                                             |
                                        C1_b |
                                        Wine |   .0836195   .1166802     0.72   0.474    -.1453296    .3125686
                                     Spirits |  -.4492333   .1093574    -4.11   0.000    -.6638137    -.234653
                                             |
                                   base_7day |  -.0012111   .0029948    -0.40   0.686    -.0070875    .0046653
                                       _cons |   2.596674   .2466687    10.53   0.000     2.112663    3.080686
--------------------------------------------------------------------------------------------------------------

.         margins i.condition##i.RET_sum_b, atmeans

Adjusted predictions                            Number of obs     =      1,086
Model VCE    : Robust HC3

Expression   : Linear prediction, predict()
at           : 1.condition     =    .1924494 (mean)
               2.condition     =    .2034991 (mean)
               3.condition     =    .2062615 (mean)
               4.condition     =     .194291 (mean)
               5.condition     =    .2034991 (mean)
               0.RET_sum_b     =    .3637201 (mean)
               1.RET_sum_b     =    .6362799 (mean)
               1.Quota_Ag~p    =    .4539595 (mean)
               2.Quota_Ag~p    =    .3001842 (mean)
               3.Quota_Ag~p    =    .2458564 (mean)
               0.gender_A2b    =    .4926335 (mean)
               1.gender_A2b    =    .5073665 (mean)
               0.educ_G2b      =    .2081031 (mean)
               1.educ_G2b      =    .7918969 (mean)
               0.parent_G3r    =    .5515654 (mean)
               1.parent_G3r    =    .4484346 (mean)
               0.seifa_qu~b    =    .2909761 (mean)
               1.seifa_qu~b    =    .7090239 (mean)
               0.NHMRCris~b    =    .3664825 (mean)
               1.NHMRCris~b    =    .6335175 (mean)
               0.B17_r         =    .5202578 (mean)
               1.B17_r         =    .4797422 (mean)
               1.C1_b          =    .3508287 (mean)
               2.C1_b          =    .3646409 (mean)
               3.C1_b          =    .2845304 (mean)
               base_7day       =    14.63444 (mean)

-----------------------------------------------------------------------------------------------------------------------
                                                      |            Delta-method
                                                      |     Margin   Std. Err.      t    P>|t|     [95% Conf. Interval]
------------------------------------------------------+----------------------------------------------------------------
                                            condition |
                     No Health Warning Label Control |   1.844773   .0881778    20.92   0.000     1.671751    2.017795
                                   DrinkWise Control |   1.834849   .0762131    24.08   0.000     1.685304    1.984394
                                           Text-Only |   2.781909   .0999385    27.84   0.000     2.585811    2.978008
                                    Text + Pictogram |   3.018032   .1025145    29.44   0.000     2.816878    3.219185
                                   Text + Photograph |   3.172467   .1036272    30.61   0.000      2.96913    3.375804
                                                      |
                                            RET_sum_b |
                                 Low (0-5 exposures) |   2.588629     .07805    33.17   0.000     2.435479    2.741778
                                High (6-8 exposures) |   2.503066   .0505315    49.53   0.000     2.403913    2.602218
                                                      |
                                  condition#RET_sum_b |
  No Health Warning Label Control#Low (0-5 exposures) |   2.146148   .1745905    12.29   0.000     1.803567    2.488728
 No Health Warning Label Control#High (6-8 exposures) |   1.672496   .0974931    17.16   0.000     1.481196    1.863797
                DrinkWise Control#Low (0-5 exposures) |   2.135485   .1434804    14.88   0.000     1.853949    2.417022
               DrinkWise Control#High (6-8 exposures) |   1.662995   .0874228    19.02   0.000     1.491455    1.834536
                        Text-Only#Low (0-5 exposures) |   2.723403   .1729241    15.75   0.000     2.384093    3.062714
                       Text-Only#High (6-8 exposures) |   2.815354   .1227497    22.94   0.000     2.574495    3.056212
                 Text + Pictogram#Low (0-5 exposures) |   2.959447   .1852334    15.98   0.000     2.595983    3.322911
                Text + Pictogram#High (6-8 exposures) |   3.051521   .1227204    24.87   0.000      2.81072    3.292322
                Text + Photograph#Low (0-5 exposures) |   2.969583   .1833178    16.20   0.000     2.609878    3.329288
               Text + Photograph#High (6-8 exposures) |   3.288443   .1238654    26.55   0.000     3.045395     3.53149
-----------------------------------------------------------------------------------------------------------------------

.
```
